# A new, Early Cretaceous carettochelyid turtle from South Korea provides insights into softshell evolution and aquatic ecology

**DOI:** 10.1186/s13358-025-00415-z

**Published:** 2025-12-08

**Authors:** Minguk Kim, Jongyun Jung, Walter G. Joyce, Jae-Il Park, Hye-Yeon Jung, Hyemin Jo, Min Huh

**Affiliations:** 1 Department of Geological and Environmental Sciences, Chonnam National University, Gwangju, 61186 South Korea; 2 Korea Dinosaur Research Center, Chonnam National University, Gwangju, 61186 South Korea; 3 Department of Earth and Planetary Sciences, Jackson School of Geosciences, The University of Texas at Austin, Austin, TX 78703 USA; 4 Department of Geosciences, University of Fribourg, Chemin du Musée 6, 1700 Fribourg, Switzerland; 5 Aging Research Groups, Honam Regional Center, Korea Basic Science Institute, Gwangju, 61751 South Korea; 6 Gwangju National Science Museum, Gwangju, 61005 South Korea; 7 Korea Heritage Service, Daejeon, 35208 South Korea

**Keywords:** *Byeoljubuchelys yeosuensis*, Early Cretaceous, Hasandong formation, Korea, *Carettochelyidae*, Paleoecology, Micro-CT

## Abstract

Here, we present a new Cretaceous carettochelyid turtle, *Byeoljubuchelys yeosuensis* gen. et sp. nov., from the Lower Cretaceous Hasandong Formation of Yeosu, Korea. A nearly complete shell and select elements of the girdles and limbs provide crucial insight into the evolution and aquatic adaptation of this family. Phylogenetic analyses place *Byeoljubuchelys yeosuensis* at the most basal position within *Carettochelyidae*. The mid-sized plastron of *Byeoljubuchelys yeosuensis* appears to be plesiomorphic for the group. The expansion of the plastron during carettochelyid evolution, especially during the Paleogene, may be an evolutionary response to increasing body size or the threat of mammalian predators. Although the shell of *Byeoljubuchelys yeosuensis* lacks distinct scute sulci, traces are apparent that are reminiscent of juvenile scutes in the extant *Carettochelys insculpta,* suggesting that scute loss was facilitated in trionychian evolution through the thickening of the epidermis. *Byeoljubuchelys yeosuensis* possesses a plesiomorphic humerus with a proximally located lateral process and a sigmoidal shaft lacking torsion, implying rowing locomotion rather than the underwater flapping seen in more recent carettochelyids. The repeated acquisition of a softshell in two clades of trionychians during the mid-Cretaceous may be related to an environmental shift towards humid climates at that time, perhaps reflecting a remarkable adaptation to freshwater ecosystems.

**Supplementary Information:**

The online version contains supplementary material available at 10.1186/s13358-025-00415-z.

## Introduction

*Carettochelyidae*, commonly known as pig-nosed turtles, represents a unique group of freshwater turtles distinguished by their softshell adaptations and aquatic ecology (Georges et al., 2008). The only extant species, *Carettochelys insculpta*, inhabits rivers, swamps, and lagoons in southern New Guinea and northern Australia (Georges et al., 2008) and stands out among extant freshwater-adapted turtles by having acquired underwater “flight” (Rivera et al., 2013). Extant pig-nosed turtles are threatened by habitat loss and overexploitation and are listed in the IUCN Red List as an endangered species (Eisemberg et al., 2018).

The earliest reported carettochelyids are *Kizylkumemys khoratensis* from the late Early Cretaceous (Aptian) of Thailand (Tong et al., 2005) and *Kizylkumemys schultzi* from the early Late Cretaceous (Cenomanian) of Uzbekistan (Nessov, 1977), but both taxa are based on partial or disarticulated specimens, respectively. As preserved, these earliest carettochelyids already resemble their modern counterparts greatly, except for being smaller, by exhibiting a strongly keeled shell with a narrow, cruciform plastron, and by retaining their carapacial scutes. This pattern mirrors the evolution of the sister group of *Carettochelyidae*, *Pan-Trionychidae*, whose earliest fossils from the late Early Cretaceous closely resemble their modern counterparts as well (Brinkman et al., 2017; Li et al., 2015a, 2015b). As the two groups are relatively different, despite being sister groups, it is apparent that many evolutionary changes between them have not been documented by the fossil record of either group, perhaps because their earliest representatives have not yet been documented. This notion is supported by molecular phylogenetic analyses, which suggest that *Carettochelyidae* diverged from *Pan-Trionychidae* as early as the Middle to Late Jurassic (Thomson et al., 2021).

In this study, we describe a new carettochelyid species, *Byeoljubuchelys yeosuensis* gen. et sp. nov., from the Lower Cretaceous Hasandong Formation (Aptian–Albian) of Yeosu, South Korea. The new species is based on a relatively complete specimen that consists of a well-preserved carapace, plastron, and associated post-cranial remains. The new species provides novel insights into the early evolution of carettochelyids and their paleoecological adaptation to the freshwater environment.

### Geological setting

**Fig. 1:**
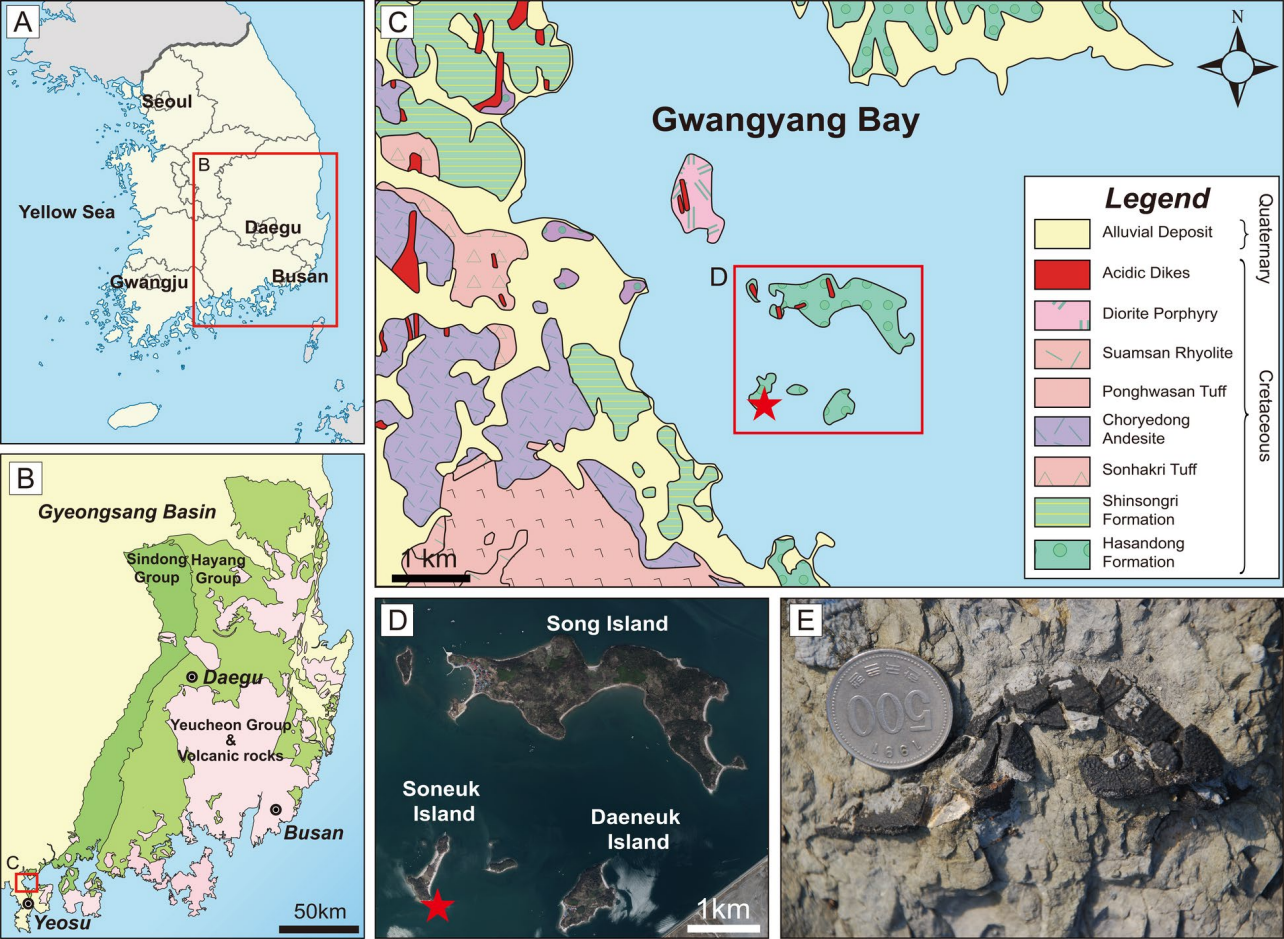
Geographical and geological settings of the type locality of *Byeoljubuchelys yeosuensis* gen. et sp. nov. **A**, Geographical map of South Korea. **B**, Geological map of Gyeongsang Basin in South Korea. **C**, Geological map of Gwangyang Bay. **D**, Aerial photograph of Soneuk Island with adjacent islands. **E**, Photographs of the holotype in situ. The red asterisks indicate fossil locality

The new carettochelyid specimen described herein was found on Soneuk Island, Yeosu, South Korea (Fig. 1). The fossil-bearing outcrop is interpreted as being part of the Hasandong Formation, from which numerous vertebrate fossils have previously been recorded, such as dinosaurs, pterosaurs, crocodiles, and turtles (references in Choi & Lee, 2017; Kim & Huh, 2018). The Hasandong Formation is part of the Sindong Group, which is subdivided into the Nakdong, Hasandong, and Jinju formations in ascending order (Chang, 1975, 1977; Choi, 1986). The Sindong Group is the lowermost group of the Gyeongsang Supergroup, which fills the Gyeongsang Basin and is overlain by the Hayang and Yucheon groups (Chang, 1975, 1977; Choi, 1986). The Gyeongsang Basin is the largest non-marine Cretaceous basin in Korea.

The Hasandong Formation comprises conglomerates, pebbly sandstones, dark gray mudstones, and reddish mudstones (Hong & Lee, 2012; Lee, 1999; Lee et al., 2023). The fossil site on Soneuk Island exposes sandstone, pebbly sandstone, dark gray mudstone, and intercalated reddish muddy sandstone. The specimen was found in an outcrop of massive, gray mudstone (Fig. 1e), which yielded several turtle shell fragments, dinosaur bone fragments, teeth, fish scales, bivalves, and gastropods.

The depositional environment of the Hasandong Formation is thought to have been an alluvial plain with low-gradient channels and floodplains (Choi, 1986; Lee et al., 2023). The paleoclimate of the Hasandong Formation was likely semi-arid. Common calcareous nodules indicate an alternation of dry and wet seasons (Kim & Pickerill, 2003; Paik & Lee, 1994). Biostratigraphically, the Hasandong Formation is interpreted as Aptian to Albian based on bivalve fossils (Yang, 1975, 1979, 1982) and mollusk fossils (Yun & Yang, 2001). That age assessment is consistent with Ion Probe, SHRIMP, and LA-MC-ICP-MS U–Pb ages (Chae, 2023; Lee, 2016; Lee et al., 2010, 2023; Sano et al., 2006). Recently, direct age dating of the nearby Jinju Yusu-ri fossil site and Hadong Janguseom Island fossil site of the Hasandong Formation indicates 117.4 ± 1.4 Ma (Aptian) and 113.4 ± 1.4 Ma (Aptian–Albian boundary), respectively (Chae, 2023). The Soneuk Island fossil site was therefore also plausibly deposited near the Albian-Aptian boundary.

## Materials and methods

The new specimen (KDRC-YS-SR-001) described herein was found by one of us (Min Huh) in 2009 in a dark-gray mudstone of the Hasandong Formation (Fig. 1c). An in situ field photograph of the specimen (Fig. 1e) shows that it was found with its posterior side up. KDRC-YS-SR-001 consists of a nearly complete carapace and plastron and associated post-cranial bones. It is now housed at the Korea Dinosaur Research Center (KDRC) at Chonnam National University, Gwangju.

KDRC-YS-SR-001 was mechanically prepared using air scribes, electronic scribes, dental scrapers, and soft brushes to remove the sediment matrix. Fragile parts and broken parts were strengthened using Paleobond and a stabilizer. Given the possibility that appendicular elements are preserved within the shell of the specimen, non-destructive micro-computed tomography (micro-CT) was chosen to observe and analyze bones hidden from view. The specimen was scanned using an Xradia 620 Versa from ZEISS (KJ318) housed at the Aging Research Group at Korea Basic Science Institute, Gwangju, South Korea. The specimen was scanned using the following parameters: voltage 140 kV, current 150 μA, power 21 W, image pixels 1532 × 968, flat panel exposure 1 s, 110 μm pixel size, 3201 projection, source filter HE3, to create 966 slices with High Aspect Ratio Tomography. The scanned data was segmented and measured using Dragonfly (version 2022.2.0.1409) from Object Research Systems (https://dragonfly.comet.tech/). The three-dimensional reconstructions were produced in orthographic view using the open-source software Blender (v. 4.1) (https://www.blender.org/). Because right peripherals Ⅷ–Ⅹ and left peripherals Ⅷ–Ⅸ detached during preparation, three-dimensional models of them were produced using Metascan (v. 2.9.9) on an Apple iPad and then digitally united with the rest of the shell. The figures in this study were created in Adobe Illustrator 2024 (v. 29.2). The slide data and 3D models were uploaded to MorphoSource and are available to the public (https://www.morphosource.org/concern/biological_specimens/000765917).

The digital scans of a specimen of *Trionyx triunguis* (UF: Herp:65522) and *Carettochelys insculpta* (TMM M18053) were obtained for comparisons. The *Trionyx triunguis* specimen (UF: Herp:65522) was accessed from MorphoSource (10.1093/biosci/biad120). The dataset is managed by David Blackburn at the Florida Museum of Natural History and was funded by oVert TCN and NSF DBI-1701714 (Blackburn et al., 2024). The Archival Resource Key (ARK) is ark:/87602/m4/384707. The *Carettochelys insculpta* specimen (TMM M18053) was scanned using a custom North Star Imaging scanner (TX-DR) housed at the High-Resolution X-ray Computed Tomography Facility at the University of Texas at Austin (UTCT) using the following parameters: voltage 150 kV, current 150 μA, 52.5 μm voxel size, 5400 projection, aluminum filter, and 1351 slices. The scan is available at https://n2t.net/ark:/87602/m4/785865 for download.

### Phylogenetic analyses

The character/taxon matrix we utilized to determine the phylogenetic affinities of *Byeoljubuchelys yeosuensis* gen. et sp. nov. is a modification of the matrices sequentially presented by Joyce (2007), Havlik et al. (2014), and Danilov et al. (2017). Character 64 (neural formula) was modified to pertain to the location of the square neural within the neural column (i.e., the neural reversal), instead of the presence of the particular neural formula found in adocusians. Two new characters were introduced based on the works of Hermanson et al. (2024) and Walther (1922) that address variation in humeral morphology, in particular the degree of twisting of the proximal versus distal ends of the humerus (character 156) and the location of the lateral process of the humerus (character 157). *Peltochelys duchastelii* and *Sandownia harrisi* were excluded relative to previous iterations of our matrix because newer studies have shown them to be completely unrelated to pan–trionychians (Evers & Joyce, 2020; Joyce & Rollot, 2020). The final matrix consists of 157 morphological characters scored for 78 taxa. All characters, character states, and scorings can be found in Supplementary Files 1 and 2.

We conducted a parsimony analysis of the full matrix using TNT 1.6 (Goloboff & Morales, 2023) with the following settings: ram increased to ‘mxram 500’, New Technology Search with Sectional search, Ratchet, Drift, and Tree fusing default options activate, and 1000 random seeds. Twenty-five characters that form morphoclines were ordered (i.e., character 7, 27, 33, 35, 54, 61, 64, 65, 68, 69, 70, 85, 89, 98, 100, 120, 133, 134, 137, 138, 142, 146, 148, 156, 157, using regular counting that starts at 1). All character state transitions were given equal weights. The hypothetical ancestor was set as the outgroup. The fossil carettochelyids *Kizylkumemys khoratensis* and *Allaeochelys lingnanica* were omitted from the final run, because initial runs identified them as rogue taxa, likely due to their fragmentary nature.

We also performed a Bayesian tip-dating analysis in MrBayes 3.2.7 (Ronquist et al., 2009), restricting the dataset to the 18 pan-trionychian taxa. Reducing taxon sampling from 78 to 18 rendered 113 characters phylogenetically uninformative. These were automatically excluded by the ascertainment bias correction, leaving 44 characters to inform the Bayesian topology. Nexus and log files can be found in Supplementary Files 3 and 4. *Adocus beatus* was designated as the outgroup. As in the parsimony analysis, all characters were equally weighted, and ordered characters were specified as morphoclines. Morphological evolution was modeled using the Lewis Mkv model with ascertainment bias correction for variable characters (Lewis, 2001). Rate heterogeneity among characters was modeled with a gamma distribution, and a subset of multistate characters was treated as ordered. An independent gamma rates (IGR) relaxed-clock model was applied, with exponential and normal priors on the rate variance and mean clock rate, respectively, to allow lineage-specific rate variation. Divergence times were estimated under the fossilized birth–death (FBD) model. Prior to speciation, extinction, and fossilization rates were weakly informative (exponential or beta distribution), the sampling proportion was set to 0.0571 (2/35), and the tree age prior followed an offset exponential distribution (minimum 120 Ma, mean 170 Ma), consistent with the stratigraphic record. Tip ages were constrained using uniform priors based on stratigraphic ranges, with extant taxa fixed at 0. Analyses were performed using Markov chain Monte Carlo (MCMC) with two runs of four chains each. The temperature parameter of the heated chains was set to 0.05, and three swaps were attempted per cycle. Chains were run for 2,000,000 generations, with trees sampled every 500 generations. The first 25% of sampled trees were discarded as burn-in. Convergence diagnostics were examined to ensure adequate mixing and stationarity. For the construction of the NEXUS file for MrBayes, we modified and followed the procedures in Evers et al. (2023).

### Systematic paleontology

*Testudines* Batsch, 1788

*Cryptodira* Cope, 1868

*Trionychia* Hummel, 1929

*Carettochelyidae* Gill, 1889

*Byeoljubuchelys yeosuensis* gen. et sp. nov. (Figs. 2, 3, 4, 5, 6, 7, 8, 9 and 10).

**Fig. 2:**
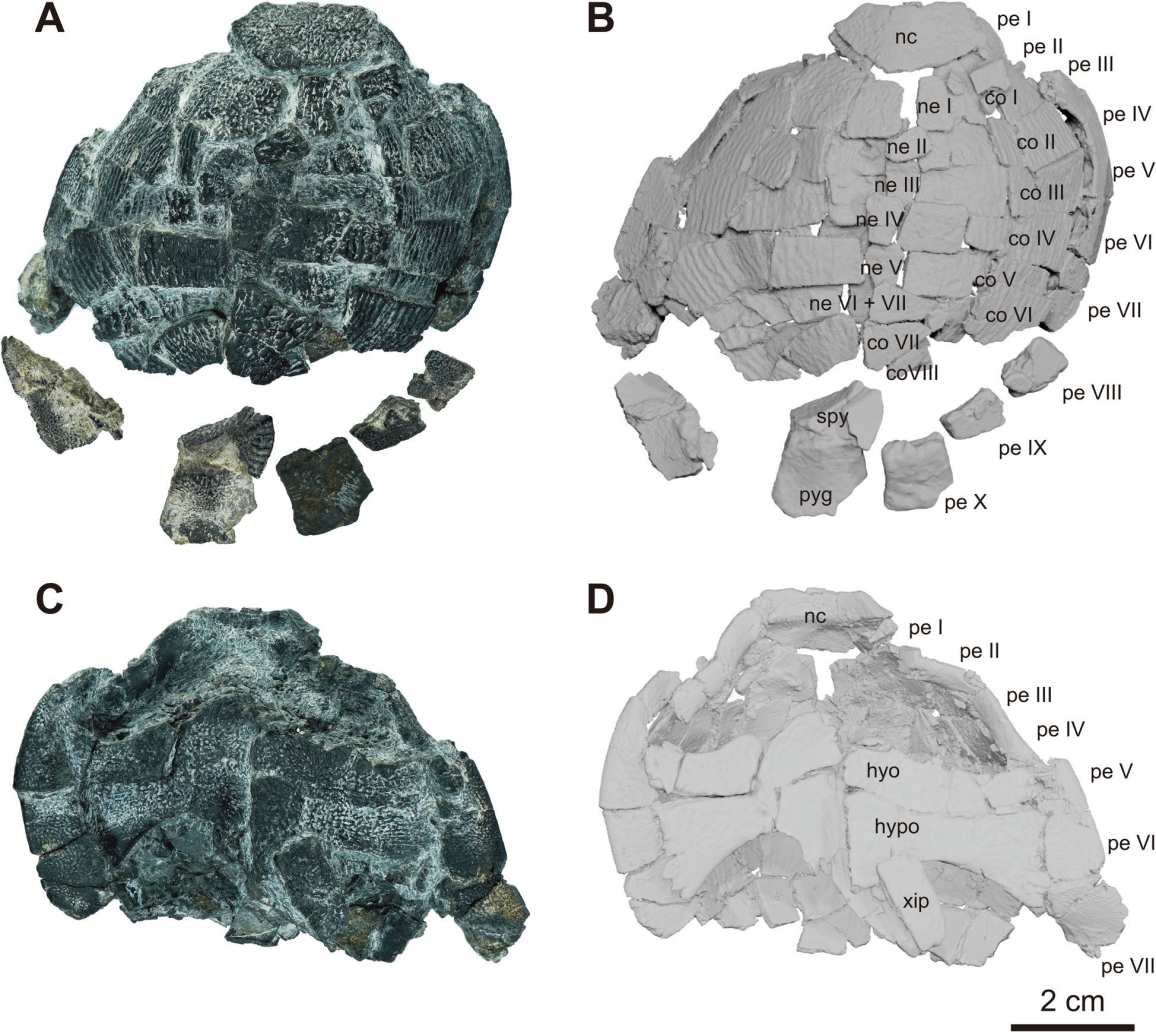
Photographs (**A**, **C**) and micro-CT generated three-dimensional models (**B**, **D**) of KDRC-YS-SR-001, holotype of *Byeoljubuchelys yeosuensis* gen. et sp. nov. **A**, **B**, shell in dorsal view. **C**, **D**, shell in ventral view. Abbreviations: co, costal; ent, entoplastron; epi, epiplastron; hyo, hyoplastron; hypo, hypoplastron; nc, nuchal; ne, neural; pe, peripheral; py, pygal; spy, suprapygal; xip, xiphiplastron

**Fig. 3:**
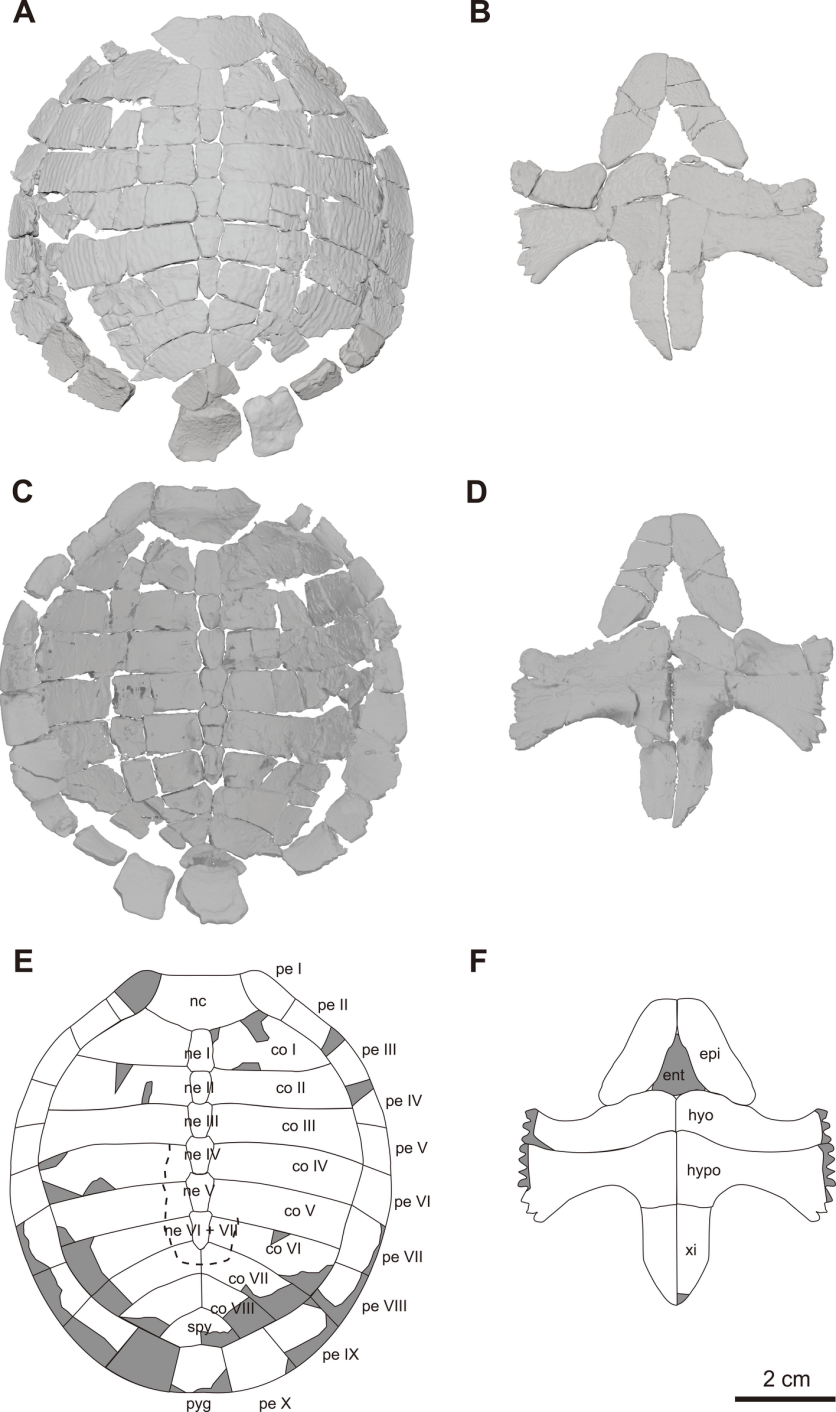
Reconstructed shell of KDRC-YS-SR-001 with preserved elements in white and lost elements in gray, holotype of *Byeoljubuchelys yeosuensis* gen. et sp. nov. Micro-CT generated three-dimensional models of the carapace in **A**, dorsal and **C**, ventral view. Micro-CT generated three-dimensional models of the plastron in **B**, ventral and **C**, dorsal view. **E**, interpretive line drawing of the carapace in dorsal view. **F**, interpretive line drawing of the plastron in ventral view. co, costal; ent, entoplastron; epi, epiplastron; hyo, hyoplastron; hypo, hypoplastron; nc, nuchal; ne, neural; pe, peripheral; py, pygal; spy, suprapygal; xip, xiphiplastron. Dashed lines indicate possible traces of scute sulci

**Fig. 4:**
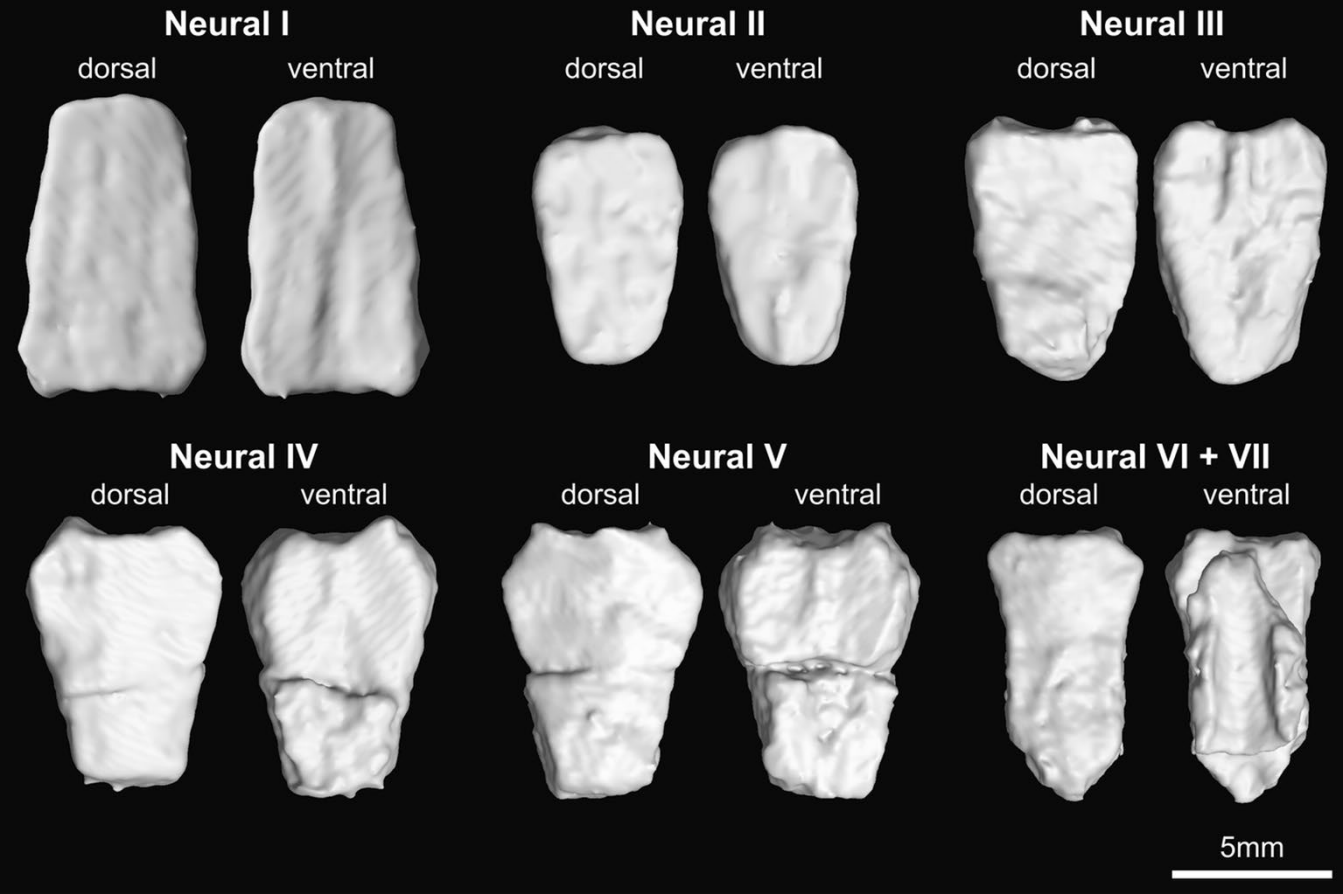
micro-CT generated 3D models of the neurals of KDRC-YS-SR-001, holotype of *Byeoljubuchelys yeosuensis* gen. et sp. nov

**Fig. 5:**
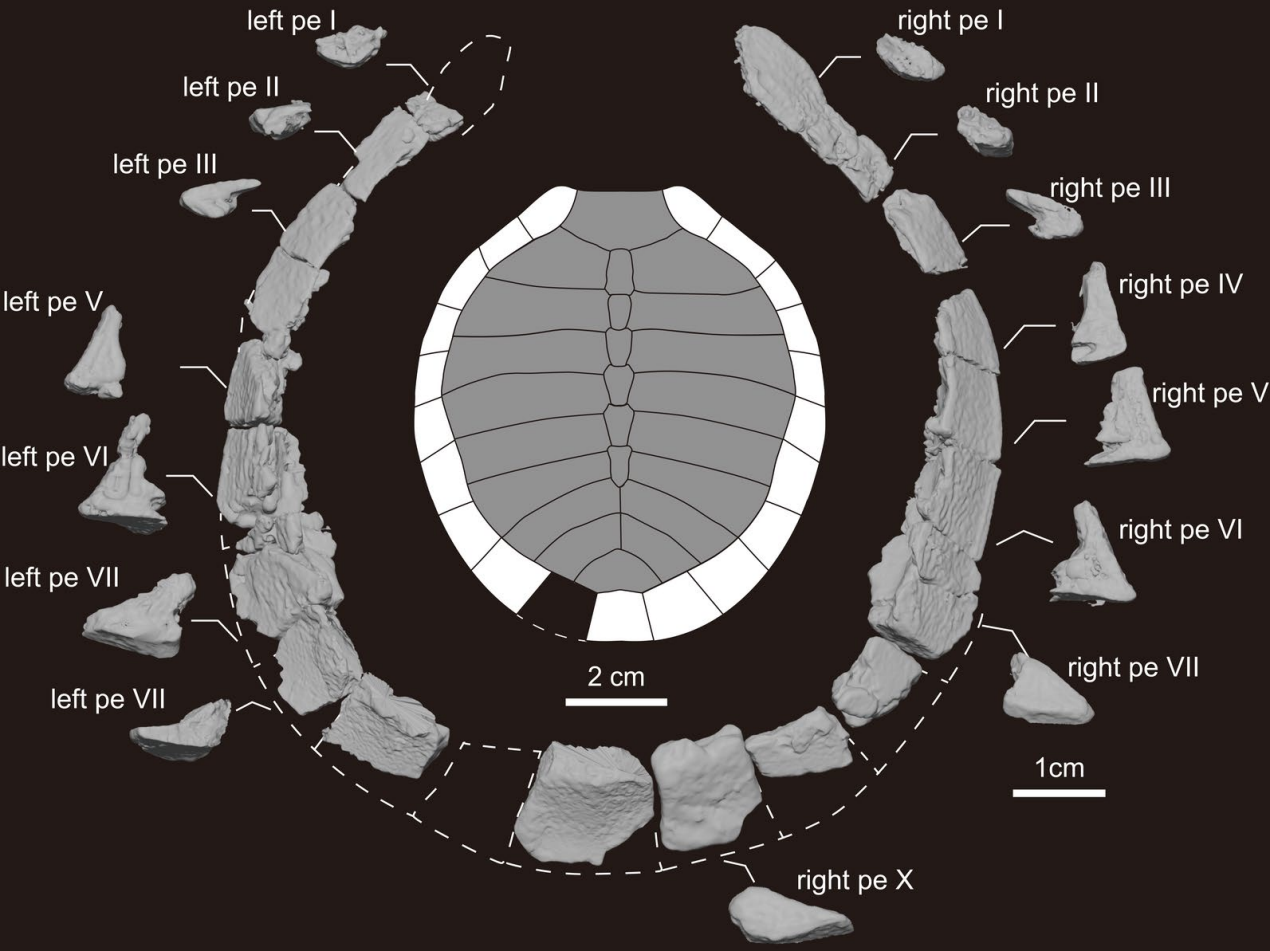
The peripherals and pygal of KDRC-YS-SR-001, holotype of *Byeoljubuchelys yeosuensis* gen. et sp. nov. Interpretive drawing of carapace in dorsal view and micro-CT generated three-dimensional models of peripherals in dorsal and posterior view. Dotted lines express the reconstructed outline of peripherals and pygal. pe, peripheral

**Fig. 6:**
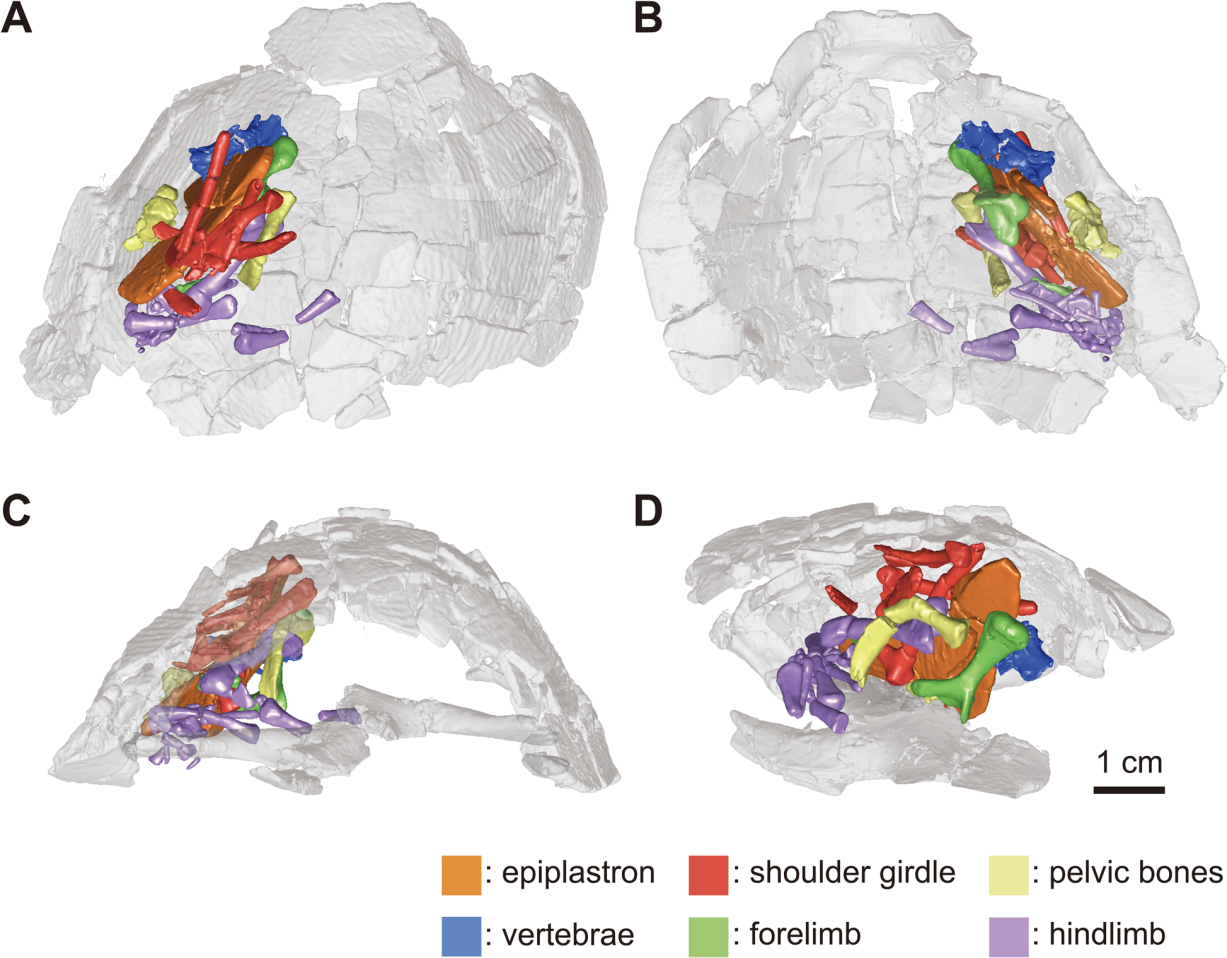
micro-CT generated three-dimensional model of KDRC-YS-SR-001, holotype of *Byeoljubuchelys yeosuensis* gen. et sp. nov., highlighting location of post-cranial and select plastral bone within the shell in **A**, dorsal, **B**, ventral, **C**, posterior, and **D**, medial view

**Holotype**—KDRC-YS-SR-001 (Korea Dinosaur Research Center, Chonnam National University, Gwangju, Republic of Korea), a partial skeleton consisting of a nearly complete shell, two cervical vertebrae, fragmented thoracic vertebrae, one sacral vertebra, both scapulae and coracoids, the left ilium and ischium, both pubes, the right humerus and ulna, the left femur, both tibiae, fibulae, and pedal remains.

**Type Locality and Horizon**—Soneuk Island, Yeosu, Republic of Korea, near the Aptian/Albian boundary, Early Cretaceous.

**Etymology**—The genus name ‘*Byeoljubuchelys’* is derived from the combination of the Korean word ‘*Byeoljubu*’, a turtle character in Sugungga, one of the epic tales of Pansori (a traditional Korean musical storytelling art), and the Ancient Greek word ‘*chelys*’ meaning turtle. The species name ‘*yeosuensis*’ is derived from Yeosu City, where the fossil was found.

Nomenclatural acts: This publication and its nomenclatural acts were registered at ZooBank prior to publication. The LSID of the publication is urn:lsid:zoobank.org:pub:9A372737-90E3-48D8-9C47-41FE225BAF6A, that of the new genus urn:lsid:zoobank.org:act:E4C87FB7-3702-471A-A05F-EA919AA94D05 and that of the new species urn:lsid:zoobank.org:act:7E11FCAF-045F-4223-B5A1-5B92C20A5047.

**Diagnosis**—*Byeoljubuchelys yeosuensis* can be diagnosed as a member of *Carettochelyidae* by the presence of 10 pairs of peripherals, a single, triangular suprapygal, a thickened pygal with an anterior groove, plastral kinesis, a triangular entoplastron, and the absence of plastral scutes. It differs from other carettochelyids by the unique combination of the following characters: neurals and pygal lacking midline keel, neural series broad and continuous, a reversal of neural orientation at neural II, vermiculated peripheral ornamentation, absence of distinct carapacial scute sulci, a lack of paired nuchal processes, a relatively broad cruciform plastron, long cervical vertebrae, and a humerus with sigmoidal shaft and lateral and medial processes at the level of the humeral head.

### Description

**Carapace**—The carapace is mostly complete, lacking the distal portions of costals VI–VIII, the anterior to middle portions of left peripheral I, the distal portion of peripherals Ⅶ–Ⅸ, and the entire left peripheral X (Figs. 2a, b and 3a, b). The articulated portion of the carapace measures about 60 mm in length along the midline, 80 mm at its greatest width at peripherals IV, and 37 mm in height. The reconstructed 3D model suggests that the carapace had a length of about 86 mm, a width of 80 mm at peripheral V, and a height of 30 mm. A carapace length of only 80 mm is striking, as the roughly coeval carettochelyids *Kizylkumemys schultzi* (Nessov, 1977) and *Kizylkumemys khoratensis* (Tong et al., 2005) are estimated to have reached a carapace length of about 250 mm and 350 mm, respectively. However, the specimen does not appear to be a hatchling, as all bones are fully developed, and fontanelles are absent. As reconstructed (Fig. 3a), the carapace has a rounded outline except for a transverse shelf formed by the nuchal and peripheral I, much as in juvenile *Carettochelys insculpta* (AMNH 85893). A minor midline keel is developed on the suprapygal, in contrast to other carettochelyids, which develop a keel along the posterior half of the shell (e.g., Carbot-Chanona et al., 2020; Harrassowitz, 1922; Hay, 1906; Nessov, 1977; Tong et al., 2010). The ornamentation on the costals generally consists of broadly rounded ridges more reminiscent of a trionychid than a carettochelyid that run parallel to the margins of the shell and become more pronounced towards the margins. The remaining elements are decorated by an indistinct pattern of low pits and vermiculated ridges. There are no fontanelles between the costals and the peripherals. Although the carapace is deformed and most components are damaged by breaks, the ornamentation on the dorsal surface of the carapace is sufficiently preserved to allow concluding that scute sulci appear to be absent on the carapace. However, faint, raised areas reminiscent of vertebral scutes can be discerned along the midline of the carapace.

The nuchal has a trapezoidal shape with a dorsally bent and anteriorly inclined surface (Fig. 3a, b). The left lateral and posterior portions are damaged. The nuchal has a length of 13.6 mm and a width of 25.6 mm, suggesting that it is twice as wide as long. The anterior margin is slightly damaged but almost flat, implying that the nuchal notch is either absent or minor. The nuchal has an inclined anterolateral contact with peripheral Ⅰ, a convex posterolateral contact with costal I, and a short posterior contact with neural I. As in pan-trionychids, there is a slight depression on the posteroventral surface of the nuchal (see 3D models) that likely accommodated the capsule surrounding the articulation of the cervical with the dorsal vertebral column, but paired processes on the ventral side of the nuchal (Meylan, 1988, Fig. 2), which are characteristic of derived carettochelyids, are absent. An indistinct pattern of low, vermiculated ridges covers the dorsal surface of the nuchal.

The neural series consists of six elements (Figs. 3a, b and 4). There is no midline keel on the neural series, which strongly contrasts with the roughly coeval *Kizylkumemys schultzi*, which exhibits a distinct keel with fin-like dorsal projections (Nessov, 1977). The neurals series resembles that of most trionychids by being continuous and relatively broad. The neurals of more derived carettochelyids are either continuous but narrow, as in *Anosteira* spp. (e.g., Adrian et al., 2020; Hay, 1906; Hutchison & Westgate, 2024; Tong et al., 2010; Zangerl, 1947) and *Allaeochelys* spp. (Dollo, 1886; Harrassowitz, 1922; Zangerl, 1947), or discontinuous, as in *Carettochelys insculpta* (Walther, 1922). As preserved, most neurals are partially overlapped by other carapacial plates (Fig. 2a, b), so minor deformation of these elements may have occurred during fossilization. Neural Ⅰ is hexagonal with short posterolateral sides and contacts costals Ⅰ and II laterally. It has a length of 9.3 mm. Neural Ⅱ is tetragonal but slightly broader anteriorly than posteriorly. It is the shortest and narrowest neural element and only contacts the costals II laterally. Neurals Ⅲ–V are hexagonal with short anterior sides. Neural Ⅳ shows a similar length to neurals Ⅵ + VII at 8.8 mm, while neural Ⅴ is slightly shorter at 8.5 mm. Neurals III–V each contact two costals laterally. Neural Ⅵ + VII has an elongate, heptagonal shape. It is 8.7 mm in length and situated between three pairs of costals. Its angular, concave lateral contacts with costals VI are highly suggestive of a fused element consisting of neurals VI and VII. The fusion of the two most posterior neural elements is a common individual variation among trionychids (Joyce, 2025) and some carettochelyids (Nessov, 1977) and should not be accorded high taxonomic value. The neurals are connected with the thoracic vertebrae ventrally. The surface ornamentation of the neurals is relatively indistinct.

All eight pairs of costals are preserved with minor damage (Figs. 2a, b and 3a, b). While costals I–VI are fully separated from one another by the neural column, costals Ⅶ have a partial, posterior midline contact with one another, and costals Ⅷ have a full midline contact. The right costal Ⅰ is damaged, but the left costal Ⅰ is relatively complete. These elements have an irregular, pentagonal outline, are oriented transversely, and are anteroposteriorly longer than the more posterior costals. The remaining costals are posteriorly curved, trapezoidal to rectangular, and have a similar medial length but widen distally. All costals have two angled medial facets for articulation with two separate neurals, except costals Ⅰ and Ⅵ, which medially contact a single neural each along straight margins. Costals Ⅰ laterally contact peripherals Ⅰ–Ⅲ, costals Ⅱ peripherals Ⅲ–Ⅳ, costals Ⅲ peripherals Ⅳ–Ⅴ, costals Ⅳ peripheral Ⅵ, costals Ⅴ peripheral Ⅶ, costals Ⅵ peripherals Ⅶ–Ⅷ, costals Ⅶ peripheral Ⅸ, and costals Ⅷ peripherals Ⅸ–Ⅹ. The ornamentation on the costals varies distinctly from medial to lateral: while the medial third is relatively smooth, the middle section has low, net-like ornamentation, and the distal section shows distinct, parallel, anteroposterior ridges.

Ten pairs of peripherals are preserved, except for most of the left peripheral I, the left peripheral X, and most of the outer rim of peripherals Ⅶ–X (Figs. 3a, b and 5). The right peripheral Ⅰ is trapezoidal and possesses a curved ventral surface. It exhibits an anterior protrusion that forms a rounded anterolateral inflection in the outline of the carapace. Peripheral Ⅱ has a rectangular shape, a curved ventral surface, and is twice as long as it is wide. Peripheral Ⅲ is trapezoidal, has a curved external rim, and mediates between the flat anterior two elements and the V-shaped bridge element. While peripherals Ⅳ–Ⅶ and Ⅹ are rectangular to square in outline, it is not possible to identify the shapes of peripherals Ⅷ–Ⅸ, as they are damaged. However, they were likely rectangular to square as well, as in other carettochelyids (e.g., Adrian et al., 2020; Hay, 1906; Rollot et al., 2025; Walther, 1922). The transition from V-shaped bridge elements to flat posterior elements occurs at peripheral Ⅷ. The angle between the dorsal and ventral surfaces of the peripheral generally increases from anterior to peripheral Ⅳ and decreases from peripheral V to X. The angle of peripherals Ⅰ–Ⅲ is about 30–40° with a rounded external rim, the anterior margin of peripheral Ⅳ is about 90°, the posterior margin of peripheral Ⅵ is about 55°, and the anterior margin of peripheral Ⅶ is about 50° with a rounded rim. Finally, the posterior margin on the posterior peripherals is 30–40° (Fig. 5). A series of indentations indicates that the osseous bridge spanned from the middle of peripherals Ⅳ to the middle of peripheral Ⅶ, as in other carettochelyids (e.g., Adrian et al., 2020; Hay, 1906; Nessov, 1977; Walther, 1922). While the posterior part of peripheral Ⅳ and all of peripheral Ⅴ articulated with the hyoplastron, all of peripheral Ⅵ and the posterior part of Ⅶ articulated with the hypoplastron. The ornamentation on the dorsal surface of the peripherals is vermiculated, but there is no ornamentation on the ventral surface except for the bridge peripherals, which have weak ventral vermiculation.

A single suprapygal is preserved, which lacks its right posterolateral aspects (Fig. 3a, b). The suprapygal appears to have been a triangular element with elongate, convex anterior rims for articulation with costals Ⅷ and three shorter posterior contacts for articulation with peripherals X and the pygal. The suprapygal thickens from its anterior to the posterior edge, reaching a maximum thickness at the low, rounded midline keel. The ornamentation consists of vermiculated ridges radiating from the keel. The pygal is trapezoidal, with a shorter anterior than posterior margin (Figs. 3a, b and 5). It flattens toward the posterior to form an acute external rim. The ventral surface curves dorsally. The midline keel almost disappears on the pygal. The dorsal surface of the pygal is inclined dorsolaterally along the keel. The ornamentation of the dorsal surface of the pygal displays slightly vermiculated ridges, while the ventral surface is unornamented, like the posterior peripherals. A visceral groove is apparent along the anterior margin of the pygal, much as in other carettochelyids (e.g., Adrian et al., 2020; Joyce et al., 2012; Rollot et al., 2025).

**Plastron**—The plastron is nearly complete, except for the entire entoplastron (Figs. 2c, d and 3c, d). The plastron is approximately three-quarters the length of the carapace. As reconstructed, the plastron measures about 64 mm along the midline and 60 mm across at the hyo-hypoplastron suture (Fig. 3c, d). The plastron has a cruciform outline with a relatively narrow bridge and triangular anterior and posterior lobes. The outline resembles that of *Anosteira* spp. (Hay, 1906; Tong et al., 2010; Zangerl, 1947) but is much broader than the starkly narrow plastron of the roughly coeval *Kizylkumemys schultzi* (Nessov, 1977). The ventral surface of the new turtle exhibits parallel ridges and vermiculations, while the dorsal surface is smooth. Scute sulci are absent. The plastron of *Byeoljubuchelys yeosuensis* resembles that of other known carettochelyids in presence of a hinge, the shape and contacts of all bones (e.g., Broin, 1977; Hay, 1906; Joyce et al., 2012; Tong et al., 2010; Zangerl, 1947).

The epiplastra are detached from the hypoplastra and situated with other post-cranial bones in the matrix between the carapace and plastron (Fig. 6). The epiplastra are elongated elements with an anteriorly curved external margin forming the lateral margin of the anterior plastral lobe (Fig. 3c, d). This elongated shape is similar to that of other carettochelyids. The epiplastra exhibit a sutural, anterior midline contact with their counterpart, extended sutural posteromedial contacts with the entoplastron, but only a blunt posterior contact with the hyoplastron, suggesting the presence of a plastral hinge between the anterior lobe and the main body of the plastron, as in other carettochelyids (e.g., Gilmore, 1931; Joyce, 2012; Tong et al., 2010; Walther, 1922). The dorsal surface of the epiplastra has a shallow fossa near the articulation with the entoplastron for attachment to the acromion process of the scapula. The ornamentation on the ventral surface of the epiplastra displays vermiculated ridges.

The hyoplastron forms a sinuous rectangle that is wider than long. Its anterior margin is undulating, with an anterior notch for articulation with the entoplastron and a lateral notch for articulation with the epiplastron (Fig. 3c, d). The hyoplastron otherwise features a broad but shallow axillary notch along its anterior rim. The posterior rim is curved following the anterior margin. The bridge section of the hyoplastron is similar to that of the hypoplastron, consisting of a series of pegs that are laterally inserted into sockets formed by the peripherals. The peripheral attachments are listed above. The ornamentation on the medial part of the hyoplastron is net-like, transitioning to a transversely parallel pattern on the lateral part.

The hypoplastron is similar in shape to the hyoplastron but is anteroposteriorly longer, resulting in longer medial contact with its counterpart and a longer bridge section (Fig. 3c, d). The posterior margin is more recurved than the anterior margin, forming a broad and deep inguinal notch that is more distinct than the axillary notch. The posterior contact with the xiphiplastron mostly consists of fine sutures, further immobilized by tooth-like projections formed by both bones. This might suggest that the present specimen is male (Joyce et al., 2012). The peripheral attachments of the hypoplastron are listed above. The ornamentation near the midline is vermiculated, while the inguinal process features fine, transversely parallel ridges.

The xiphiplastra form the posterior plastral lobe with the posterior part of the hypoplastra (Fig. 3c, d). The xiphiplastra jointly form the shape of a shield, as the lateral margins of their anterior halves are arranged parallel to one another, while those of their posterior halves converge towards the midline. The posterior portions of the xiphiplastron slightly curve upwards toward the carapace. An anal notch appears to be lacking. Although disarticulated from the hypoplastron, the xiphiplastron connects to the hypoplastron via a serrated suture supported by tooth-like projections formed by both bones. The ornamentation of the xiphiplastron is relatively smooth.

**Fig. 7:**
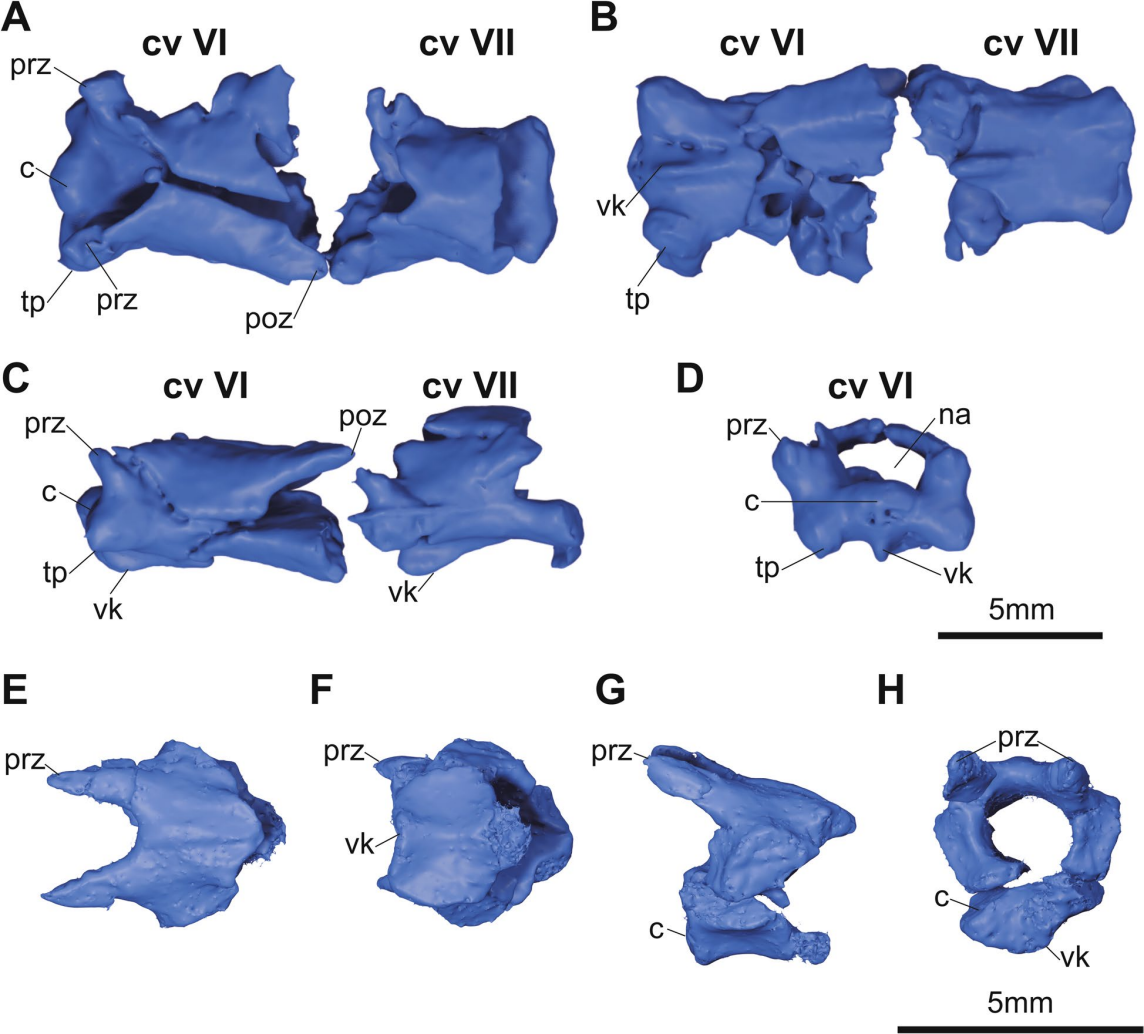
micro-CT generated three-dimensional models of cervical (**A**–**D**) and sacral (**E**–**H**) vertebrae of KDRC-YS-SR-001, holotype of *Byeoljubuchelys yeosuensis* gen. et sp. nov. in **A**, **E**, dorsal, **B**, **F**, ventral, **C**, **G**, left lateral, and **D**, **H**, anterior view. c, centrum; cv, cervical vertebra; poz, postzygapophysis; prz, prezygapophysis; tp, transverse process; vk, ventral keel

### Vertebrae

Two cervical vertebrae are preserved (Fig. 7a–d). They are heavily fractured by dorsoventral compression, but the anterior one is relatively well preserved nonetheless. Neural spines are not developed on either cervical vertebra. The anterior articulation of the anterior of the two elements is simple convex, while the posterior articulation is double concave. The anterior articulation of the posterior element, by contrast, is double convex, but the posterior one is double concave as well. This strongly suggests, by comparison to extant *Carettochelys insculpta,* that the two vertebrae represent cervicals Ⅵ and Ⅶ (Walther, 1922). A distinct ventral keel is preserved along the anterior halves of both vertebrae. The keel is more ventrally pronounced on cervical VII. The centra of both elements are notably wider than tall.

Remains of the thoracic vertebrae can be found within the shell, but these are too fragmentary to provide meaningful anatomical insights.

One sacral vertebra is preserved with a damaged posterior articulation (Fig. 7e–h). The centrum has a concave anterior articulation with a broken posterior surface. The dorsal surface is smooth and posteriorly inclined with distinct prezygapophyses. The ventral surface is wide and exhibits a shallow ventral keel. This is different from *Carettochelys insculpta,* which has a smooth ventral surface.

**Fig. 8:**
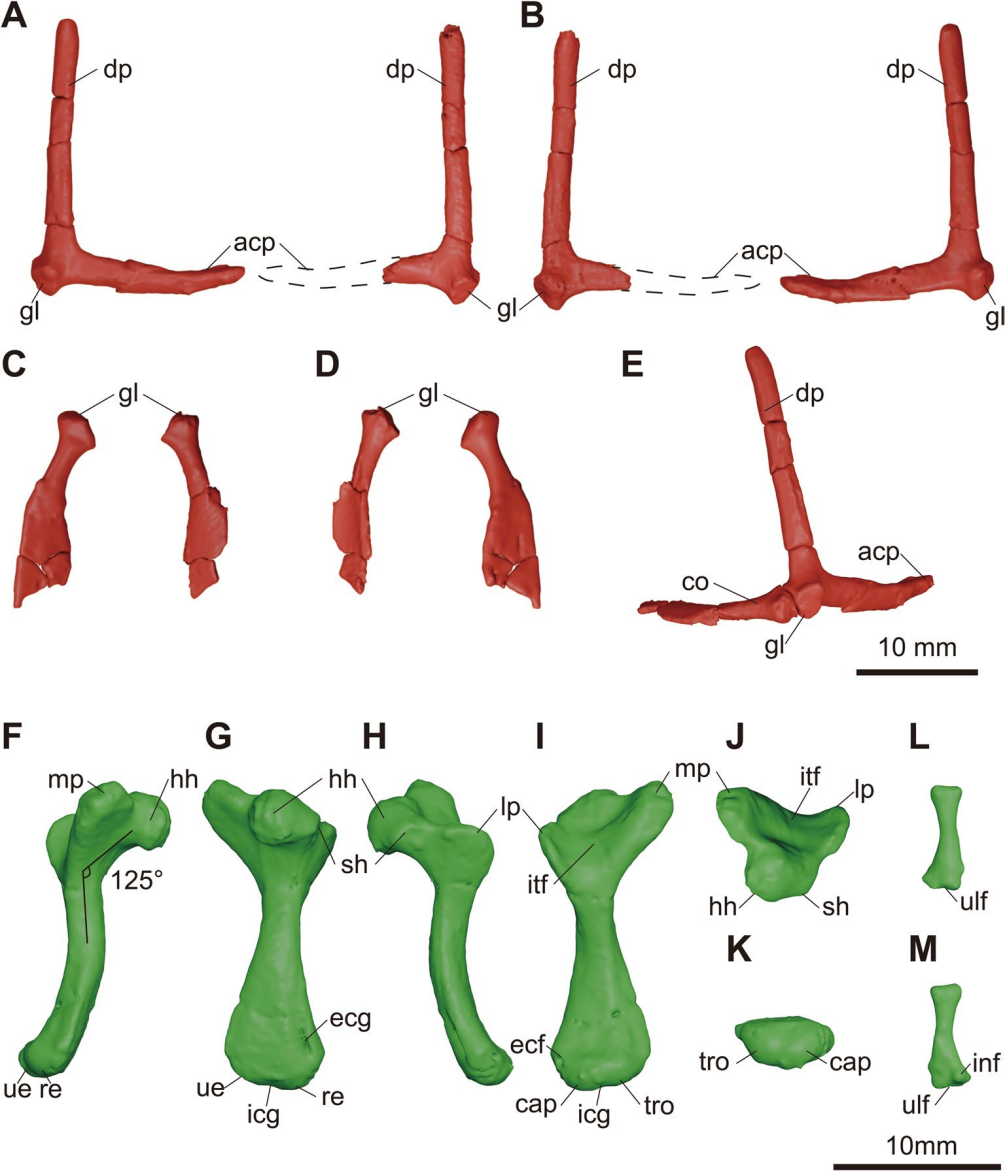
micro-CT generated three-dimensional models of the shoulder girdles (**A**–**E**) and forelimbs (**F**–**M**) of KDRC-YS-SR-001, holotype of *Byeoljubuchelys yeosuensis* gen. et sp. nov. Scapulae in **A**, anterior, **B**, posterior view. Coracoids in **C**, dorsal, and **D**, ventral view. Reconstructed left shoulder girdle in **E**, anterolateral view. Right humerus in **F**, ulnar, **G**, capitular, **H**, radial, **I**, intertubercular, **J**, proximal, and **K**, distal view. Right ulna in **L**, capitular and **M**, intertubercular view. Stippled lines express reconstructed outline of scapula. acp, acromion process; cap, capitellum; co, coracoid; dp, dorsal process; ecf, ectepicondylar foramen; ecg, ectepicondylar groove; gl, glenoid; hh, humeral head; icg, intercondylar groove; inf, intermedium facet; itf, intertubercular fossa; lp, lateral process; mp, medial process; re, radial epicondyle; tro, trochlea; ue, ulnar epicondyle; ulf, ulnar facet

### Limbs and girdles

The pectoral girdles consist of the disarticulated scapulae and coracoids (Fig. 8a–e). The left scapula is preserved with the dorsal process disarticulated and a crack in the medial third of the acromion process. The right scapula is preserved with an incomplete acromion process. The scapula is bifurcated into a dorsal and an acromion process. The dorsal process is long and cylindrical and stands at an angle of about 95° to the acromion process, as in *Allaeochelys crassesculpta* and *Carettochelys insculpta* (Joyce et al., 2012; Walther, 1922). It dorsoventrally becomes circular to oval. The acromion process has an oval cross-section at its base but is distally expanded, flattened, and slightly upturned, as in *Carettochelys insculpta* (Walther, 1922). A glenoid neck is not present. The scapula forms the glenoid fossa together with the coracoid. Even though the left coracoid is highly fragmented, the right coracoid is relatively well preserved. The coracoid expands distally to form a flat paddle. The distal outline of the coracoid is unknown.

The right humerus and ulna are the only preserved elements of the forelimb (Fig. 8f–k). The humerus is cracked at the base of the proximal and distal metaphyses, but the overall shape is well-preserved. The humerus has a sigmoidal shaft with a projecting humeral head and a curved diaphysis. This contrasts with *Carettochelys insculpta*, which exhibits a relatively straight diaphysis (Hermanson et al., 2024). The proximal portion has a humeral head, a faint shoulder, and proximally positioned lateral and medial processes. The humeral head is oval-spherical in shape, and it is angled from the shaft at about 125°. The indistinct humeral shoulder is confluent with the humeral head and separates the head from the lateral process. The lateral process is smaller and lower than the medial one. The base of the lateral process is located at a similar level to the base of the humeral head, which resembles the humerus of trionychids, but is different from the humerus of *Carettochelys insculpta*, where the lateral process is displayed distally (Hermanson et al., 2024). The medial process is well-developed and has a rounded end. It extends proximally higher than the level of the humeral head, again as in trionychids, but not as extensively as in *Carettochelys insculpta* (Hermanson et al., 2024). The intertubercular fossa separates the lateral and medial processes. The humeral shaft widens distally, forming the ulnar and radial epicondyles, which are separated by a low intercondylar groove. The radial epicondyle is located more distally than the ulnar epicondyle but has a similar low and rounded shape. The capitellum and trochlea are not distinct, similar to juvenile *Carettochelys insculpta* (Hermanson et al., 2024). A well-developed ectepicondylar groove is present on the distal portion of the capitular surface, which leads to the ectepicondylar foramen, which exists next to the capitellum. An enclosed ectepicondylar canal is typically absent in trionychids, but present in *Carettochelys insculpta* (Hermanson et al., 2024).

The ulna is completely preserved (Fig. 8l, m). The distal portion is much wider than the proximal portion. The proximal part shows a rounded articular surface for the humerus. The shaft has a subspherical cross-section. Four articulation sites are apparent on the distal end: an elongate facet at the side of the shaft for articulation with the pisiform, two distal articulations with the ulnare and intermedium, and a small facet on the other side of the shaft for articulation with the radius. A broad contact of the ulna with the pisiform is not known in trionychids and is present in *Carettochelys insculpta* (Walther, 1922), but its adaptive significance, if any, is unclear to us.

**Fig. 9:**
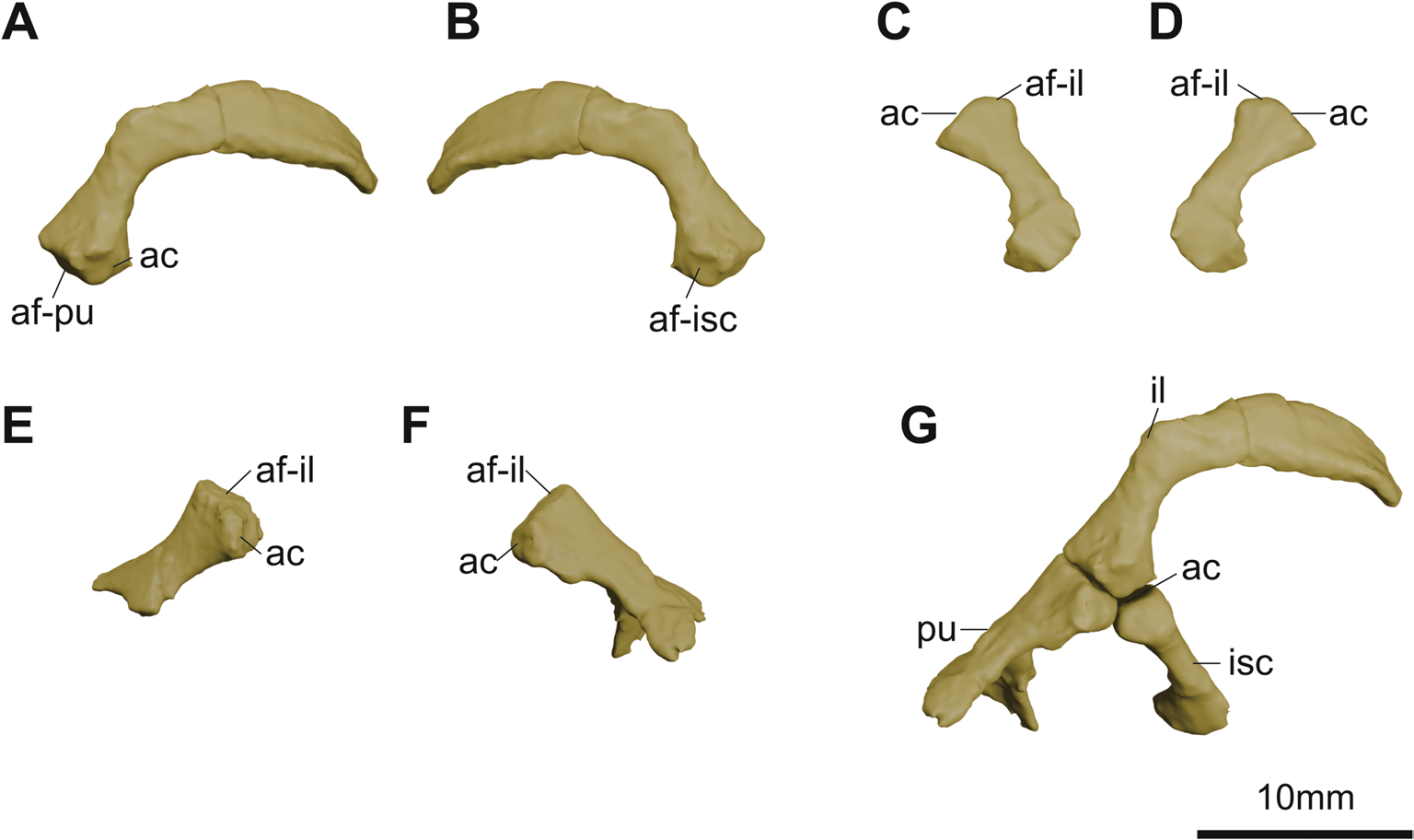
micro-CT generated three-dimensional models of the pelvic girdles of KDRC-YS-SR-001, holotype of *Byeoljubuchelys yeosuensis* gen. et sp. nov. Left ilium in **A**, lateral and **B**, medial view. Right ischium in **C**, dorsal and **D**, ventral view. **E**, left, and **F**, right pubis in left and right lateral view, respectively. Reconstructed pelvic girdles in G, lateral view. ac, acetabulum; af-il, articular facet for ilium; af-isc, articular facet for ischium; af-pu, articular facet for pubis; il, ilium; isc, ischium; pu, pubis

The pelvic girdle is represented by the left ilium, the left ischium, and the pubes (Fig. 9). The ilium is a curved, L-shaped element with a thick, robust acetabular region and an ilial process that is cylindrical at its base and striated and flattened dorsally, as in extant trionychids, but with a more distinct mid-shaft kink. By contrast, the ilial process of *Carettochelys insculpta* has a less pronounced kink and is strongly recurved dorsally (Walther, 1922). The acetabular surface is oriented anterolaterally. The ischium is rectangular, arching inward, and widens medially. It has a robust acetabular surface. The medial portion shows damage, so it is not clear if a medial process was present. The anterior portion of the pubes is damaged. The right pubis is composed of the medial process, the lateral process, and the acetabular process. The left pubis only consists of the acetabular process. The medial process broadens medially and anteriorly with a shallow depression on the posterior margin. The anterior rim of the pubioischiadic foramen might be horizontally flat, considering the preserved posterior rim of the medial process. The lateral process projects laterally downward and is smaller and narrower than *Carettochelys insculpta* (Walther, 1922). It cannot be determined whether a gap or a cartilaginous epipubis existed between the pubes.

**Fig. 10:**
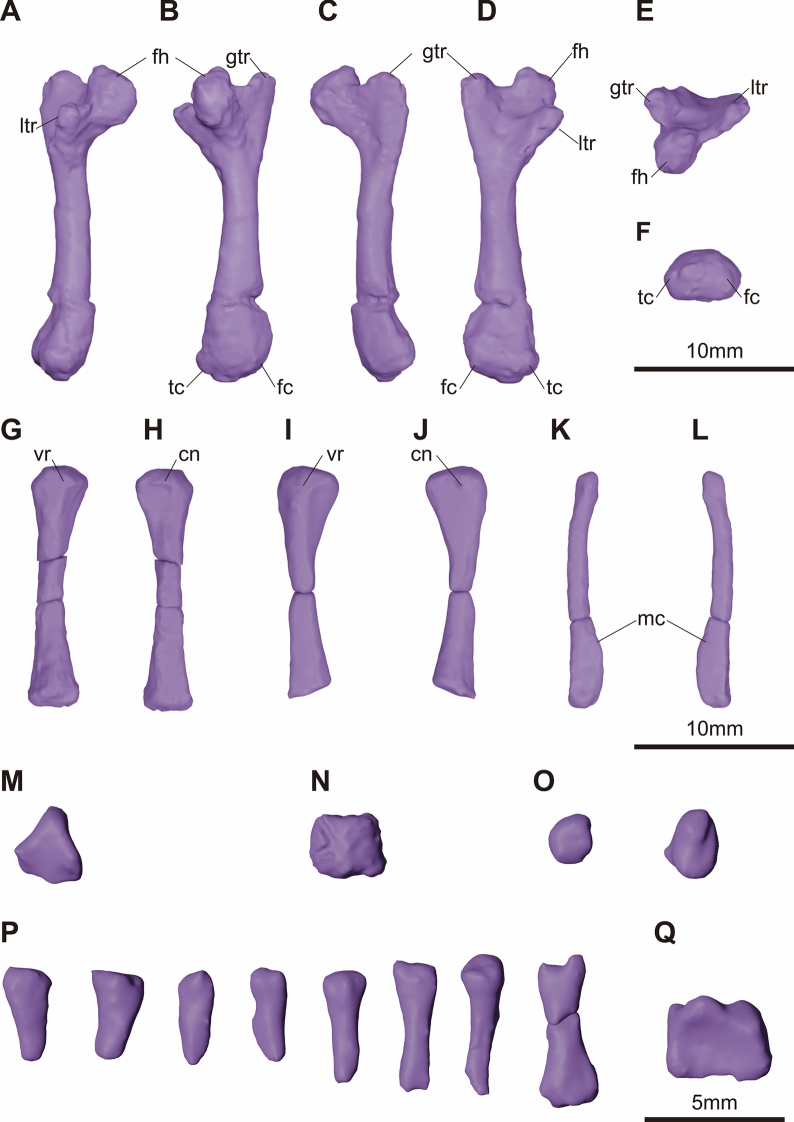
micro-CT generated three-dimensional models of the hindlimb components of KDRC-YS-SR-001, holotype of *Byeoljubuchelys yeosuensis* gen. et sp. nov. Left femur in **A**, tibial, **B**, capitular, **C**, fibular, **D**, intertrochanter, **E**, proximal, and **F**, distal view. Left tibia in **G**, ventral and **H**, dorsal view. Right tibia in **I**, ventral and **J**, dorsal view. Left fibula in **K**, ventral and **L**, dorsal view. **M**, astragalus. **N**, calcaneum. **O**, tarsal components. **P**, unidentified digits and metatarsals. **Q**, metatarsal V. cn, cnemial crest; fc, fibula condyle; fh, femoral head; gtr, greater trochanter; ltr, lesser trochanter; mc, medial crest; tc, tibia condyle; vr, ventral ridge

The hind limbs are represented by the left femur, the right and left tibiae, and much of the right and left fibulae (Fig. 10a–l). The femur is slightly damaged at its proximal and distal ends but otherwise preserved almost fully intact (Fig. 10a–f). Its shaft is straight compared with that of the humerus. This is similar to *Carettochelys insculpta* (Walther, 1922), but differs strongly from trionychids, which have a sinusoidal shaft. The proximal end comprises the femoral head, the greater trochanter, and the lesser trochanter. The femoral head is an oblong sphere, as in extant trionychids, not a rounded sphere, as in *Carettochelys insculpta* (Walther, 1922). The greater trochanter is wide and flat in shape and only partially offset from the femoral head. Its proximal end is similar in height to the femoral head, which contrasts with those of extant trionychids and *Carettochelys insculpta* (Walther, 1922)*,* which exhibit more elongated greater trochanters that are taller than the femoral head. The lesser trochanter is shorter and thicker than the greater trochanter. The femoral shaft widens distally less than that of the humerus. Two condyles are ventrally developed on the distal end and separated by a slight groove. The fibular condyle is larger than the tibial condyle. The iliofemoralis ridge is developed on the intertrochanteric side and the posterior femoral fossa is open posteroventrally.

The tibiae are the thinnest at mid-shaft and become thicker and wider proximally and distally (Fig. 10g–j). The proximal part has a flat articular site with a slight depression on the medial side. The cross-section of the proximal end is triangular. The ventral ridge is located on the ventral surface of the proximal end, and the cnemial crest is on the dorsal surface of the proximal part. The fibulae are rod-shaped with triangular distal portions (Fig. 10k, l). While the proximal part is thin and cylindrical with a spherical cross-section, the distal part is a subtriangular cross-section with a medial ridge from the medial to the lateral part. The joint knob for the tarsus is rounded and protrudes over the concave surface.

The preserved components of the left pes are identified as the astragalus, the calcaneum, the hooked fifth metatarsal, and a small number of unidentified metatarsals, tarsals, and phalanges (Fig. 10m–q). The astragalus remains attached to the tibia along a proximal groove (Fig. 10m). The calcaneum is relatively flat, pentagonal, and slightly pointed at the bottom (Fig. 10n). A projection of the calcaneum attaches to a shallow distal groove of the astragalus. The two likely tarsals are flat and oval in shape. However, it is hard to identify their particular positions within the foot (Fig. 10o). The unidentified metatarsals and phalanges of the digits are mostly broken, except for the fifth metatarsal (Fig. 10p, q). However, it suggests that the foot was elongated. The fifth metatarsal shows a large, flat quadrangle shape, but the articular process with the fifth digit is less developed than that of *Carettochelys insculpta* (Walther, 1922) (Fig. 13q).

## Phylogenetic results

We performed two analyses, parsimony and Bayesian tip-dating, to determine the phylogenetic affinities of *Byeoljubuchelys yeosuensis* gen. et sp. nov. The parsimony analyses yielded six trees with 430 steps, a consistency index of 0.447, and a retention index of 0.808. In the strict consensus and the 50% majority rule tree (see Supplementary Files 5 and 6), *Byeoljubuchelys yeosuensis* is placed in *Carettochelyidae* without autapomorphies. *Pan-Trionychia* is supported by six common synapomorphies [64, square neural shifted from neural Ⅰ to neural Ⅱ (0–1); 90, plastral midline sinuous (0–1); 92, extragular scutes gained (1–0) 108, articulation between cervical Ⅳ and Ⅴ changed from concave to convex (1–0); 109, articulation between cervical Ⅴ and Ⅵ changed from concave to convex (1–0); 110, articulation between cervical Ⅳ and Ⅶ changed from concave to convex (1–0)]. *Trionychia* is supported by 10 common synapomorphies [23, fusion of premaxillae present (0–1); 24, foramen intermaxillaris gained (0–2); 28, lateral contact between vomer and pterygoid lost (0–1); 44, medial contact of pterygoids lost (0–1); 65, number of peripherals reduced to 10 pairs (1–2); 75, ligamentous bridge present (0–1); 89, plastral scutes lost (0–2);103, ventral keels on the posterior cervical vertebrae lost (1–0); 134, short flippers present (0–1); 142, manual claws reduced to three (0–2)]. *Carettochelyidae*, which excludes *Byeoljubuchelys yeosuensis*, consists of a fully unresolved polytomy supported by three unambiguous synapomorphies [64, location of the square neural is changed from neural Ⅱ to neural Ⅰ (1–0); 139, paired ventral processes of nuchal present (0–1); 147, distinct carapacial keel gained (0–1)]. The American *Anosteira* clade is supported by one common synapomorphy [140, partially or completely divided vertebral Ⅰ (0–1)]. *Allaeochelys* + *Carettochelys* clade is supported by four additional synapomorphies [17, quadratojugal maxilla contact present (0–1); 137, fossa behind quadrate enlarged (1–2); 141, width of posterior plastral lobe expanded (1–0); 150, foramen posterius canalis caroticus interni shifted posterior to parabasisphenoid (0 or 1–2)].

**Fig. 11:**
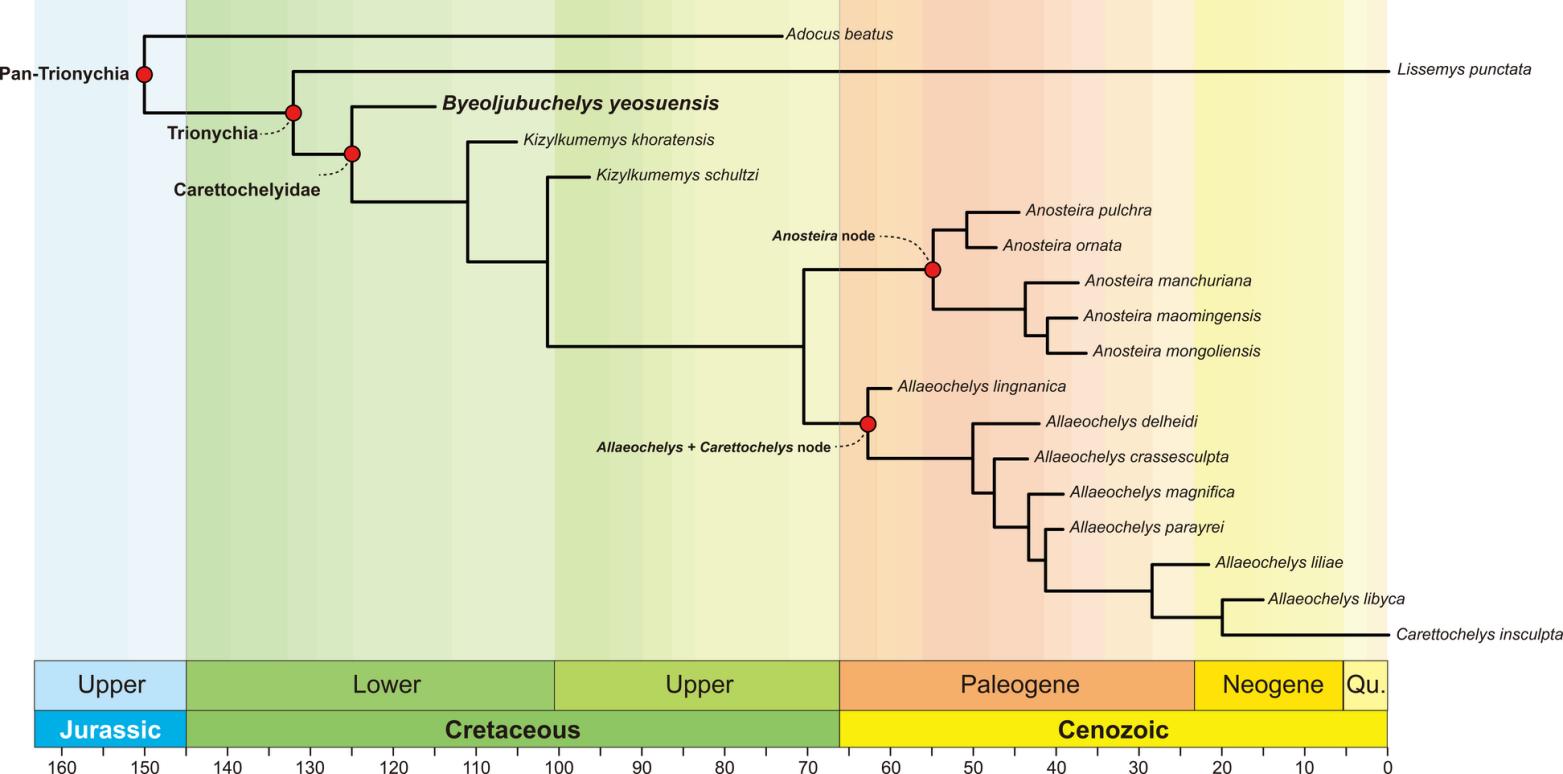
Result Markov Chain Monte Carlo (MCMC) analyses from the Bayesian tip-dating phylogenetic analysis with scaled geological time

The half compatibility tree with 50% credible nodes retrieved from the Bayesian analysis is provided in Fig. 11. *Carettochelyidae* is retrieved as a monophyletic relative of *Lissemys punctata*. Within *Carettochelyidae*, *Byeoljubuchelys yeosuensis* forms the earliest branching taxon, followed by *Kizylkumemys khoratensis* and *Kizylkumemys schultzi*. A more derived clade within *Carettochelyidae* comprises “*Allaeochelys*” *lingnanica*, and what we informally term the *Anosteira* node and the *Allaeochelys* + *Carettochelys* node. Within the *Anosteira* node, the North American *Anosteira ornata* and *Anosteira pulchra* are separated from all Asian taxa. The result is similar to previous phylogenetic results (Adrian et al., 2020; Havlik et al., 2014; Joyce, 2014). Within the *Allaeochelys* + *Carettochelys* node, Eocene *Allaeochelys* form a basal polytomy, but Miocene *Allaeochelys* (i.e., *Allaeochelys liliae* and *Allaeochelys libyca*) are more closely related to the extant *Carettochelys insculpta*.

## Discussion

### Plastral expansion in* Carettochelyidae*

Previous studies have proposed an evolutionary trend within *Carettochelyidae* characterized by progressive plastral expansion from basal to more derived taxa (Havlik et al., 2014; Joyce, 2014). This trend includes an anteroposterior elongation of the plastral bridge and lateral expansion of the anterior and posterior lobes. However, the conclusion that early carettochelyids uniformly possessed narrow plastral morphologies is challenged through the discovery of *Byeoljubuchelys yeosuensis* gen. et sp. nov. The plastron of *Byeoljubuchelys yeosuensis* gen. et sp. nov. is notably broader than that of the mid-Cretaceous *Kizylkumemys schultzi*, featuring a longer plastral bridge, a widely open axillary and inguinal notch, and expanded epiplastra and xiphiplastra. These features render its plastron more comparable to *Anosteira* spp.

**Fig. 12:**
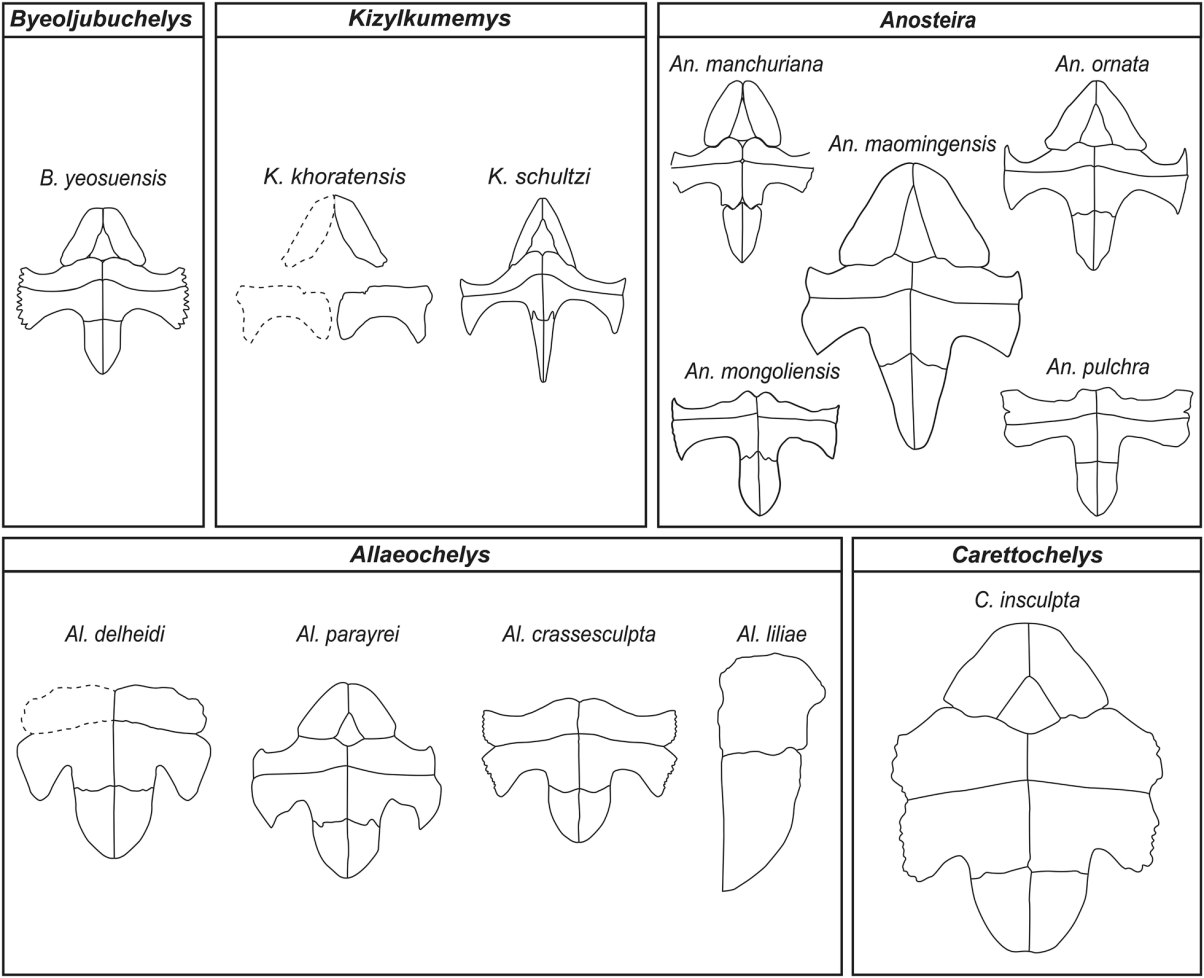
The plastra of *Carettochelyidae* in the ventral view, highlighting evolutionary trends. *Kizylkumemys khoratensis* redrawn from Tong et al. (2005). *Kizylkumemys schultzi* redrawn from Nessov (1977). *Anosteira manchuriana* redrawn from Zangerl (1947). *Anosteira ornata* redrawn from Hay (1906). *Anosteira maomingensis* redrawn from Tong et al. (2010). *Anosteira mongoliensis* redrawn from Gilmore (1931). *Anosteira pulchra* redrawn from Clark (1932). *Allaeochelys delheidi* drawn based on STUS 14069 (https://saladelastortugas.usal.es/libro/1479/). *Allaeochelys crassesculpta* redrawn from Harrassowitz (1922). *Allaeochelys parayrei* redrawn from Broin (1977). *Allaeochelys liliae* redrawn from Carbot-Chanona et al. (2020). *Carettochelys insculpta* redrawn from Joyce (2014). The interpreted drawings are not scaled, and dashed lines delineate reconstructed parts

Interestingly, the mid-Cretaceous *Kizylkumemys khoratensis*, which has previously been interpreted as having a narrow plastron similar to *Kizylkumemys schultzi* (Tong et al., 2005), may in fact possess a more intermediate morphology as well. Based on the preserved elements, which include the epiplastron and hypoplastron (Tong et al., 2005, Fig. 2a, c), *Kizylkumemys khoratensis* possesses a broadened epiplastron with a curved anterolateral margin, a blunt posterior process, and a long hypoplastral bridge (Fig. 12). The plastron of *Kizylkumemys khoratensis* thus appears to be more similar to that of *Byeoljubuchelys yeosuensis* than *Kizylkumemys schultzi* which exhibits a markedly narrow plastron, typified by a short bridge, sharply angled anterior lob, and slender epiplastron (Nessov, 1977). *Kizylkumemys schultzi* furthermore differs from both *Kizylkumemys khoratensis* and *Byeoljubuchelys yeosuensis* by exhibiting a distinct keel with elongate projections. Considering that *Kizylkumemys khoratensis* and *Kizylkumemys schultzi* are never retrieved in phylogenetic analyses as immediate sister taxa (Adrian et al., 2020; Havlik et al., 2014; Joyce, 2014), we find it plausible that *Kizylkumemys*, as currently circumscribed, may not be monophyletic and that *Kizylkumemys schultzi* represents a highly derived condition within the carettochelyids, possibly reflecting a temporary or local trend towards pronounced secondary plastral reduction.

In conclusion, these comparisons among Cretaceous carettochelyids yield two key insights: (1) while an overall trend of plastral expansion exists within the clade, early members may have possessed broader plastra than previously thought; and (2) the extremely narrow plastron of *Kizylkumemys schultzi* is likely a specialized condition, rather than the plesiomorphic trait of the group.

The plastron in turtles performs multiple functions, including providing ventral protection, anchoring the pectoral and pelvic girdles, and reinforcing the lateral carapacial margins (Richmond, 1964). Among aquatic turtles, such as members of *Trionychidae*, *Cheloniidae*, and *Chelydridae*, but also Mesozoic macrobaenids and sinemydids, reduced, cruciform plastra are frequently observed. These reductions are generally interpreted as hydrodynamic adaptations that also facilitate vertical limb excursion and expansion of the ventral adductor musculature (Zhu, 2011).

Within *Pan-Trionychia*, basal taxa, such as *Basilochelys* (Tong et al., 2009) and *Sinaspideretes* (Tong et al., 2014), exhibit broad plastra akin to those of *Adocusia*. In contrast, basal, mid-Cretaceous members of both *Pan*-*Trionychidae* [e.g., *Perochelys* spp. (Brinkman et al., 2017; Li et al., 2015a)] and *Carettochelyidae* [e.g., *Kizylkumemys* spp. (Nessov, 1977; Tong et al, (2005)] have been interpreted as having reduced plastra as well, which presumably reflect aquatic adaptations, but, as discussed above, secondary expansion is apparent for numerous trionychians, including carettochelyids, cyclanorbines, and plastomenids (Girard et al., 2024; Havlik et al., 2014; Hutchison, 2009; Joyce, 2014).

Since the plastron plays a key role in stabilizing the carapace, its enlargement may represent a structural adaptation to accommodate increased body mass. The secondary expansion seen in carettochelyids may be related to increasing body size as *Byeoljubuchelys yeosuensis* and *Anosteira* spp. are relatively small-bodied and possess intermediately sized plastra, whereas the more derived *Allaeochelys* spp. exhibit a medium-sized body with a broad plastron, and extant *Carettochelys* combines a large body size with a highly expanded plastron. Incidentally, this trend does not hold true for trionychids, where the largest representatives plesiomorphically retain reduced plastra while notably smaller forms, in particular cyclanorbines and plastomenids, develop expanded plastra (Havlik et al., 2014; Joyce, 2014; Pérez-García & Smith, 2021; Vitek, 2012).

Alternatively, secondary plastral expansion in *Carettochelyidae* may also be tied to shifts in predator–prey dynamics following the K-Pg boundary. After the massive extinction, new selective pressures from small-bodied mammalian predators emerged. During the Paleocene and Eocene, for instance, members of *Baenidae* developed enlarged, thickened parietals and supraoccipitals (Lyson & Joyce, 2009), and plastomenid softshell turtles, as noted above, evolved more ossified and broadened plastra (Lyson et al., 2021) that are interpreted as defensive responses to mammalian predation (Lyson & Joyce, 2009; Lyson et al., 2021). Given their relatively soft shells, carettochelyids may have experienced similar selective pressures, which could have contributed to the re-expansion of the plastron in more derived members of the clade.

### Softshell evolution of the* Carettochelyidae*

An evolutionary trend toward scute reduction from basal to more derived taxa has been documented within *Carettochelyidae* (Havlik et al., 2014; Joyce, 2014). In the plastron, scutes initially persisted along the medial region (Nessov, 1977), but eventually disappeared entirely after the Cretaceous. A similar pattern of reduction is evident in the carapace, where scutes decreased from the peripheral margins inward, culminating in their complete loss after the Paleocene (Adrian et al., 2020; Tong et al., 2010; Young & Chow, 1962). Traditionally, the presence of scutes in fossil turtles has been inferred from the occurrence of scute sulci. Accordingly, shells lacking sulci have been interpreted as being entirely covered by skin, as in extant trionychids. However, ontogenetic observations of *Carettochelys insculpta* complicate this interpretation. Juvenile individuals of this extant turtle display cervical and vertebral scutes, which gradually diminish during ontogeny and do not imprint sulci onto the shell (Zangerl, 1959). These findings demonstrate that sulci are not necessarily reliable indicators for the presence of scutes in carettochelyids and open the possibility that scutes may have been present in a fossil turtle even when the sulci are absent.

*Byeoljubuchelys yeosuensis* gen. et sp. nov. provides additional insight into this issue. Its carapace exhibits slightly raised ridges on the costals IV–VII (Fig. 3a, b) that resemble the outline of the cervical and vertebral scutes observed in *Kizylkumemys* spp. and *Anosteira* spp. (Adrian et al., 2020; Cheng, 1961; Clark, 1932; Nessov, 1977; Zangerl, 1959). A transverse ridge in *Byeoljubuchelys yeosuensis* located on the last neural is morphologically closer to the vertebral sulcus of *Anosteira* than to the more linear ridges of *Kizylkumemys* (Adrian et al., 2020; Nessov, 1977; Tong et al., 2010), indicating a possible homology with scute boundaries. We thus suspect that *Byeoljubuchelys yeosuensis* possessed reduced scutes that did not form impressed scute sulci, much as in the extant, juvenile *Carettochelys insculpta*.

Based on ontogenetic and fossil evidence, the diversity of scute and sulcus configurations in *Carettochelyidae* can be categorized into four morphological states: (1) plesiomorphic scutation with fully impressed sulci (e.g., *Kizylkumemys* spp., most *Anosteira* spp.), (2) reduced scutation with partially impressed sulci (no information on softshell) (*Anosteira maomingensis*), (3) no impressed sulci (i.e., a softshell) and floating scutes as described above (*Byeoljubuchelys yeosuensis*, subadult *Carettochelys insculpta*), and (4) a softshell without scute and sulci (*Allaeochelys* spp., adult *Carettochelys insculpta*). This variability implies that scutes and softshell can coexist independently of sulci in carettochelyids and warrants a re-examination of previously assigned softshell conditions in other fossil taxa.

The absence of distinct sulci on the carapace and plastron of *Byeoljubuchelys yeosuensis* suggests that the softshell condition in carettochelyids may have originated as early as the Early Cretaceous. Importantly, the observed coexistence of scutes and softshell in both various ontogenetic and taxonomic contexts provides critical insight into the evolution of softshell morphology within *Trionychia*. While trionychids uniformly lack sulci across both basal and derived taxa, carettochelyids display a broader range of scute and sulcus conditions. Given that scutes are not known to have re-evolved in any group of turtles once lost (Joyce, 2007), the presence of scutes in carettochelyids likely represents a retention of the ancestral condition. This suggests that the common ancestor of *Trionychia* may have had thickened dermal tissue overlaying the shell, causing scutes to float above the bone surface without producing sulci, as exemplified by *Byeoljubuchelys yeosuensis*. In *Trionychidae*, this configuration appears to have further evolved toward the complete loss of scutes and sulci, as observed in Early Cretaceous taxa such as *Perochelys* spp. (Brinkman et al., 2017; Li et al., 2015a, 2015b). In contrast, carettochelyids likely underwent phylogenetic variations in skin thickness and scute retention. Although definitive fossils of the last common ancestor of *Trionychia* remain unknown, the presence of well-defined sulci in basal pan-trionychians, such as *Basilochelys macrobios* and *Sinaspideretes wimani* (Tong et al., 2009, 2014), indicates that scutes were fully developed and in direct contact with the shell surface at an early stage. In this context, *Byeoljubuchelys yeosuensis* may represent a transitional form in which dermal softening began to obscure sulci boundaries without a complete loss of scutes.

The acquisition of softshell in *Trionychia* may have been influenced by the expansion of a fluvio-lacustrine ecosystem through the warming of East Asia in the Early Cretaceous. Trionychids from the late Early Cretaceous already displayed some diagnostic characteristics, like the radical decrease of shell ossification, the reorganization of the plastron, and the apparent loss of the scute (Brinkman et al., 2017; Li et al., 2015a, 2015b). The softshell itself, one of the most distinct characters of trionychids, not only provides advantages, such as facilitating cutaneous breathing and thermoregulation, but also causes water evaporation and heat loss (Plummer et al., 2005; Ultsch et al., 1984). In the Early Cretaceous, when the first basal trionychids and carettochelyids appeared, global warming expanded the range of the Hadley circulation, pushing the humid climate zone in the mid-latitude area across the globe, including Asia (Hasegawa, 2012). The humid zone in the mid-Cretaceous increased the extent of fluvio-lacustrine environments, and may have provided an aquatic habitat for turtles with soft shells, as suggested by Nakajima et al. (2017). The independent acquisition of softshell at this time may thus have been influenced by climate change.

### Scapular and humerus morphologies and aquatic adaptation

Turtles inhabit diverse environments, including oceans, streams, lakes, ponds, and dry land, and their shoulder girdles, pelvic girdles, and limbs often present shapes that are indicative of the environment they inhabit. Fortunately, *Byeoljubuchelys yeosuensis* gen. et sp. nov. preserves elements of the pectoral girdle and long bones, offering valuable insights into the ecology of early carettochelyids at the base of their evolutionary history.

**Fig. 13:**
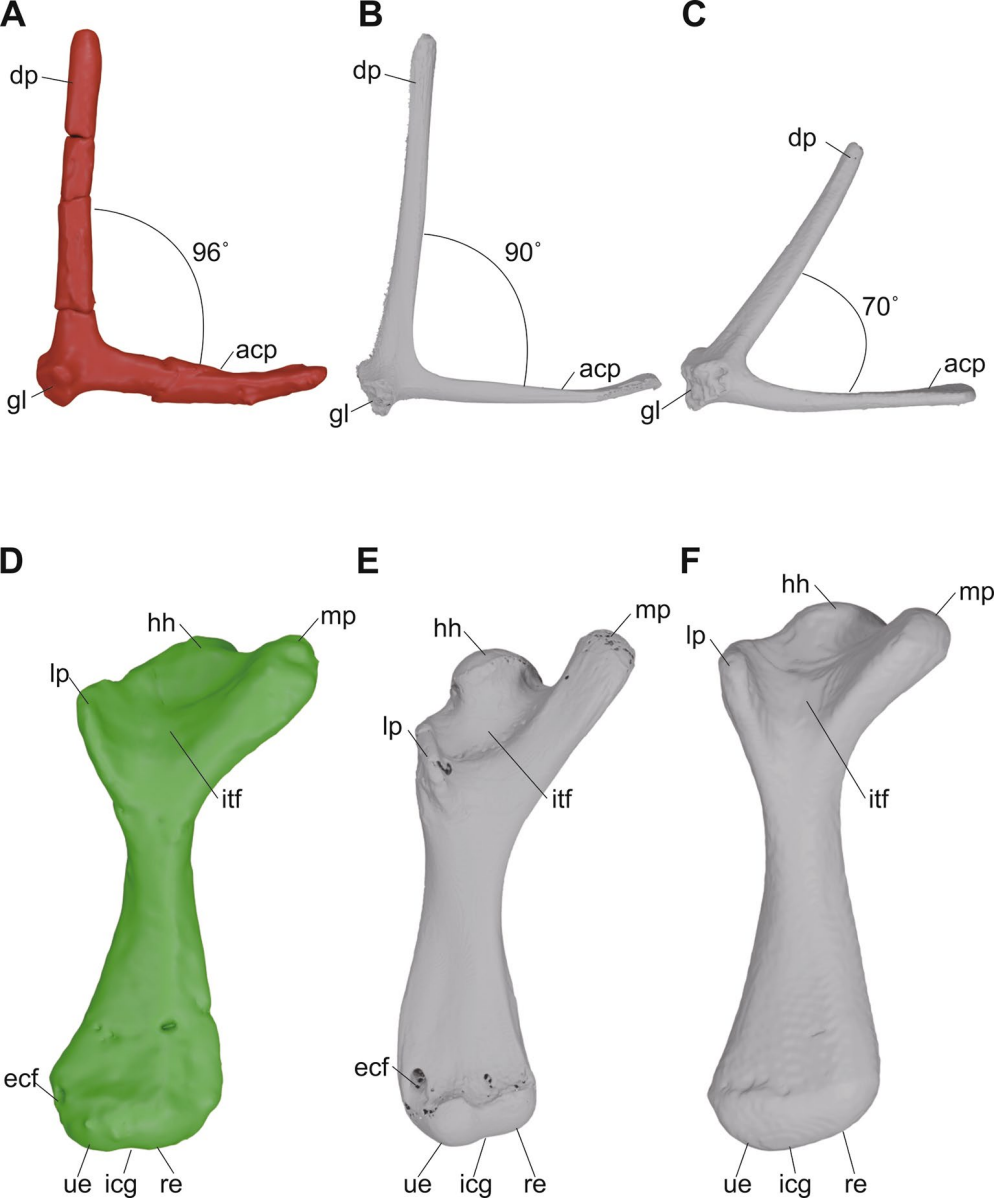
Comparison of the left scapula (**A**–**C**) and right humerus (**D**–**F**) of **A**, **D**, *Byeoljubuchelys yeosuensis* gen. et sp. nov, **B**, **E**, *Carettochelys insculpta* (TMM M18053), and **C**, **F**, *Trionyx triunguis* (UF/H 65522) in anterior (**A**–**C**) and intertubercular view (**D**–**F**). Not to scale. acp, acromion process; dp, dorsal process; ecf, ectepicondylar fossa; gl, glenoid; hh, humeral head; icg, intercondylar groove; itf, intertubercular fossa; lp, lateral process; mp, medial process; rc, radial condyle; uc, ulnar condyle

In a seminal paper, Depecker et al. (2006) conducted a geometric morphometric analysis of the shoulder girdle of extant turtles. Although their analysis grouped turtles by ecological categories (terrestrial, marine, and highly aquatic freshwater), it did not specifically identify morphological correlates of habitat. Notably, their “highly aquatic” group consisted largely of trionychians, excluding other aquatic taxa like *Hydromedusa tectifera*, suggesting the grouping may reflect phylogeny rather than pure ecology. The scapula of *Byeoljubuchelys yeosuensis* exhibits an angle of about 95° between the dorsal process and acromion process of the scapula (Fig. 13a). The same angle is about 90° in *Carettochelys insculpta* (Fig. 13b) and about 70° in *Trionyx triunguis* (Fig. 13c). Although Depecker et al. (2006) note that “highly aquatic” turtles have a low scapular angle, this measure mostly reflects the doming of a turtle, with low-domed turtles having a low angle and high-domed turtles a high angle. The 95° we observe for *Byeoljubuchelys yeosuensis* thus only indicates that this taxon had a relatively high domed shell, like many other aquatic turtles. Depecker et al. (2006) otherwise note that aquatic turtles have relatively elongate acromion processes, slim scapular and acromion processes, and a slim, elongate coracoid. In that regard, the slim pectoral girdle of *Byeoljubuchelys yeosuensis* appears to confirm aquatic habitat preferences but does not indicate how well-adapted to aquatic environment this turtle was.

The underwater flying locomotion of *Carettochelys insculpta* is distinct among extant freshwater turtles, including trionychids (Krahl, 2021; Krahl & Werneburg, 2023; Rivera et al., 2013). The synchronous motion of the fore flippers is similar to that of sea turtles (Walther, 1922), but evolved convergently. Detailed kinematic studies, however, show that *Carettochelys insculpta* combines rowing and underwater flying and that there is a difference in flapping style between *Carettochelys insculpta* and *Caretta caretta* (Krahl, 2021; Krahl & Werneburg, 2023; Rivera et al., 2013). The humeri of *Carettochelys insculpta* and the fossil *Allaeochelys crassesculpta* are distinct in that their distal parts are twisted relative to the proximal parts along the long axis of the shaft (Walther, 1922). This twisting, which is lacking in marine turtles, results in an anterolateral orientation of the forelimbs, outwardly facing palms, and hyperextension of the elbow (Hermanson et al., 2024; Walther, 1922). However, the humerus of *Byeoljubuchelys yeosuensis* exhibits an angle of only approximately 10° between the proximal and distal ends.

In the evolution of the turtle lineage, the angle between the humeral head surface (proximal parts) and the plane of the ulnar and radial condyle (distal parts) initially decreased from a right angle to 0°–10° (Hermanson et al., 2024). In particular, the humerus of *Eunotosaurus africanus*, a plausible early stem turtle, shows a torsion of 90° (Cox, 1969). The twisting is reduced to 10°–30° in basal Triassic testudinatans, such as *Proganochelys quenstedti*, and almost disappears to 0°–10° at the base of crown turtles, but then secondarily reappears in *Carettochelys insculpta* (Hermanson et al., 2024). The humerus of *Byeoljubuchelys yeosuensis* shares additional, plesiomorphic characteristics with extant softshell turtles, including a similar level of the medial process with the humeral head, proximally located lateral process, and a sigmoidal humeral shaft (Fig. 13d–f). Extant trionychids, like most freshwater aquatic turtles, show an anteroposterior rowing locomotion (Rivera et al., 2013). We thus conclude that the plesiomorphic humerus of *Byeoljubuchelys yeosuensis* indicates that this Early Cretaceous carettochelyid used a rowing motion when swimming, different from the extant *Carettochelys insculpta*, which, in return, suggests that underwater flight evolved at a later time in carettochelyid evolution.

## Conclusion

We describe a new carettochelyid turtle, *Byeoljubuchelys yeosuensis* gen. et sp. nov.*,* based on an almost complete shell associated with select remains of the girdles and limbs from the Early Cretaceous (Aptian-Albian) Hasandong Formation of the southern Korean Peninsula. *Byeoljubuchelys yeosuensis* is easily recognized to be a carettochelyid by the presence of only 10 peripherals, a single pygal, and an intermediately sized kinetic plastron with enlarged epiplastra and entoplastron. It differs from other carettochelyids by exhibiting a trionychid**-**like surface texture, a rectangular second neural, presence of slight keel only on the suprapygal, only faint impressions of scutes, and a plesiomorphic humerus with a proximally placed lateral process and a sigmoidal shaft that lacks torsion. Phylogenetic analyses retrieve *Byeoljubuchelys yeosuensis* as the earliest branching carettochelyid within *Carettochelyidae*. The intermediately sized plastron of *Byeoljubuchelys yeosuensis* appears to be a plesiomorphy for *Carettochelyidae*. The highly reduced plastron of *Kizylkumemys schultzi* is thus likely an apomorphic specialization. The expansion of the plastron in later carettochelyids either accommodated a larger size or provided better protection against mammalian predators. The absence of distinct sulci in combination with the presence of raised areas resembling juvenile scutes in *Carettochelys insculpta* raises the possibility that *Byeoljubuchelys yeosuensis* possessed scutes that floated above the shell, perhaps due to a thickening of the epidermis, hinting at a possible evolutionary mechanism for the development of the softshell seen in trionychians. The synchronous evolution of a softshell in two separate clades of *Trionychia* during the Early Cretaceous may be related to environmental changes at that time towards a more humid climate. The delicate pectoral girdle and untwisted humerus of *Byeoljubuchelys yeosuensis* suggest that this turtle swam using a plesiomorphic rowing locomotion, highlighting a different swimming mechanism at the base of carettochelyid evolution.

## Supplementary Information

Additional file 1.

Additional file 2.

Additional file 3.

Additional file 4.

Additional file 5.

Additional file 6.

## Data Availability

The slide data and 3D models were uploaded to MorphoSource and are available to the public (https://www.morphosource.org/concern/biological_specimens/000765917).

## Abbreviations

AMNH: American Museum of Natural History, New York, USA
KDRC: Korea Dinosaur Research Center, Chonnam National University, Gwangju, South Korea
STUS: Universidad de Salamanca, Salamanca, Spain
UF: Florida Museum of Natural History, Gainesville, USA
TMM: Texas Vertebrate Paleontology Collections, The University of Texas at Austin, Austin, Texas, USA
