# Supplementary figures and images for: A new, Early Cretaceous carettochelyid turtle from South Korea provides insights into softshell evolution and aquatic ecology
Source: Swiss J Palaeontol. 2025 Dec 8;144(1):75. doi: 10.1186/s13358-025-00415-z (PMC12686080; doi:10.1186/s13358-025-00415-z)

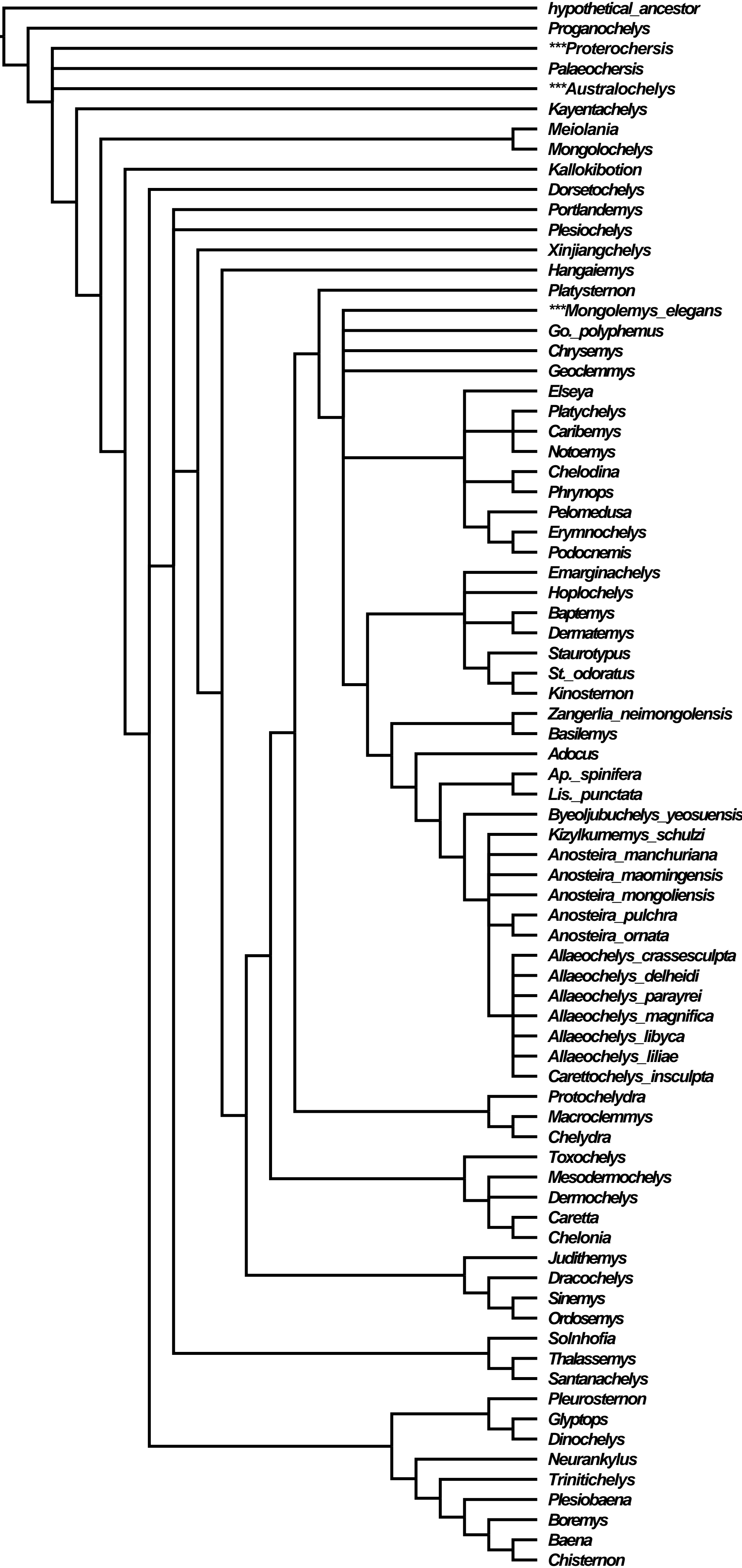

Supplement: Supplementary file 5 — Additional file 5. [file 13358_2025_415_MOESM5_ESM.pdf]

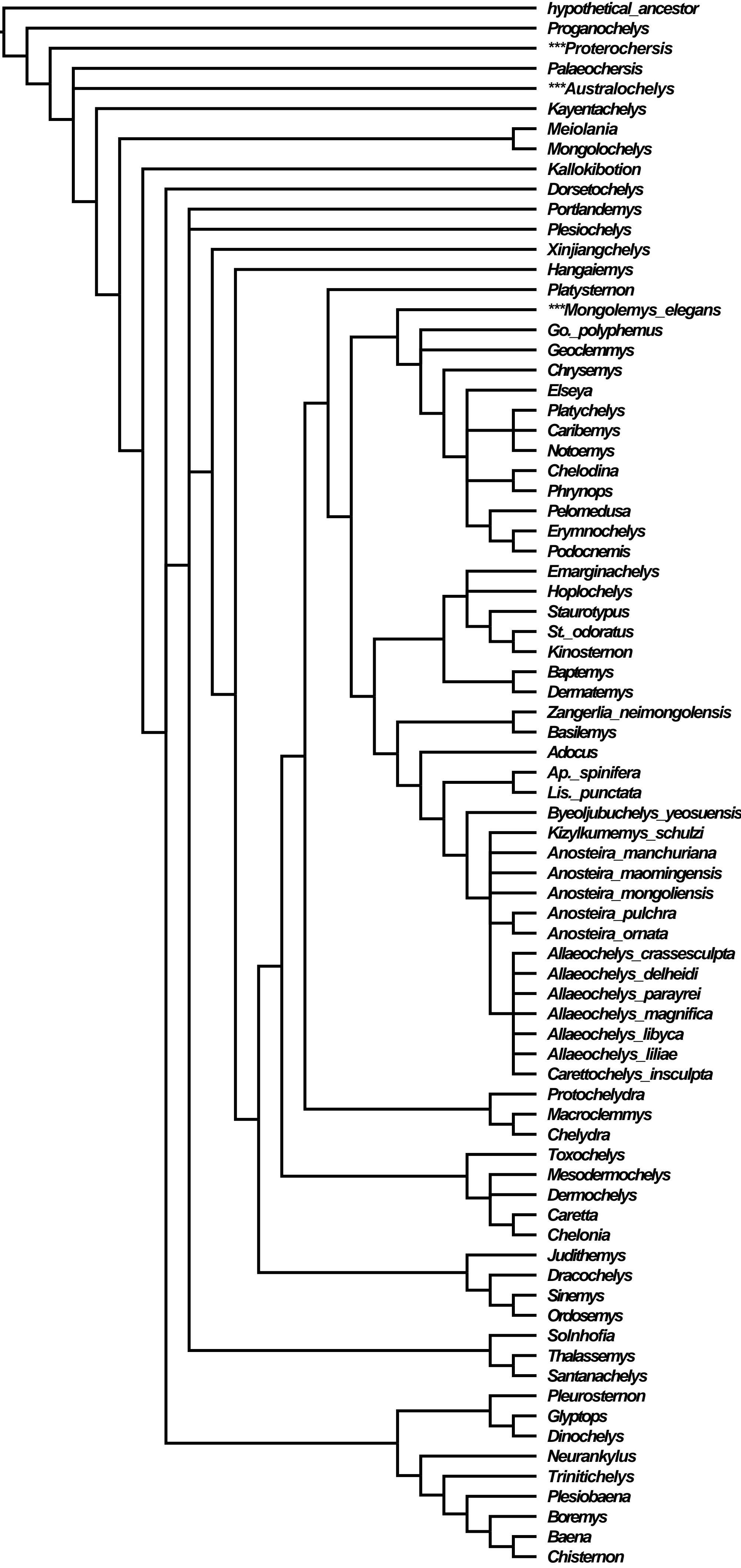

Supplement: Supplementary file 6 — Additional file 6. [file 13358_2025_415_MOESM6_ESM.pdf]
